# Cancer-associated Fibroblast-like Cells Promote Osteosarcoma Metastasis by Upregulation of Phosphoserine Aminotransferase 1 and Activation of the mTOR/S6K Pathway

**DOI:** 10.7150/ijbs.109169

**Published:** 2025-06-20

**Authors:** Liwen Feng, Guangqin Xiao, Yuting Chen, Ting Ye, Li Fan, Yuxiu Xie, Ting Mei, Lei Wang, Jingjing Ge, Chengzhi Ye, Jing Chen

**Affiliations:** 1 Cancer Center, Union Hospital, Tongji Medical College, Huazhong University of Science and Technology, Wuhan 430022, China.; 2 Department of Oncology, Beijing Chao-Yang Hospital, Capital Medical University, Beijing 100020, China.; 3 Department of Oncology, First Affiliated Hospital of Zhengzhou University, Zhengzhou 450052, China.; 4 Department of Medical Oncology, State Key Laboratory of Oncology in South China, Guangdong Provincial Clinical Research Center for Cancer, Sun Yat-sen University Cancer Center, Guangzhou 510060, China.; 5 Department of Pediatrics, Renmin Hospital of Wuhan University, Wuhan 430060, China.

**Keywords:** bone tumor, lung metastasis, migration, metabolomics, serine metabolism, mammalian target of rapamycin

## Abstract

Metastasis remains a major obstacle limiting the survival of patients with osteosarcoma (OS). Cross-talk between cancer-associated fibroblasts (CAFs) and OS cells has been found to facilitate metastasis, although the effects of CAFs on OS cell metabolism are poorly understood. Here, conditioned medium from OS cells was used to activate CAF-like cells, which was found to promote OS migration and the epithelial-mesenchymal transition. Metabolomics analysis showed that treatment of OS cells with CAFs-conditioned medium significantly altered the levels of phosphoserine aminotransferase 1 (PSAT1), a key serine synthase. CAF-induced OS cell migration was inhibited by PSAT1 knockdown by siRNA, and PSAT1 was found to promote migration through the PI3K/mTOR/S6K pathway. The influence of CAFs on OS metastasis was blocked by PSAT1 knockdown and mTOR inhibitors *in vitro* and *in vivo*. In conclusion, the findings suggest that PSAT1 and the mTOR/S6K pathway have the potential as targets for preventing OS metastasis.

## Introduction

Osteosarcoma (OS) represents 35% of primary bone malignancies and is associated with poor prognosis 1, 2. The current standard treatment strategy for OS is surgery combined with high-intensity chemotherapy 3. However, 30-40% of OS cases still develop postoperative metastases, resulting in a long-term survival rate below 20% and contributing to an unfavorable prognosis 4, 5. Despite continuing exploration of treatments for metastatic OS, the treatment approaches remain limited, necessitating further research into metastatic OS.

Recent findings have shown that tumor progression is controlled not only by the tumor cells themselves but also by their interaction with stromal cells 6, 7. Cancer-associated fibroblasts (CAFs) predominate (>50%) in the stromal cell population; these are spindle-shaped fibroblasts that are located within or adjacent to the tumor and are primarily derived from tumor-activated fibroblasts or mesenchymal stem cells (MSCs) 8, 9. The CAF switch to a malignant phenotype has been linked with tumorigenic behavior in OS cells, together with the promotion of the epithelial-mesenchymal transition (EMT), angiogenesis, maintenance of stemness, and the ability to modify immune system components 9. CAFs also induce changes in the extracellular matrix (ECM), leading to alterations in the physicochemical properties of the tumor and changes in the tumor cytoskeleton and the expression of genes linked to matrix remodeling 10. In addition, CAFs have secretory functions, releasing various factors that modulate tumor cells, including transforming growth factor-β (TGF-β) and the interleukins IL-6 and IL-8, amongst others, which provide supportive drivers for tumor metastasis 11-13.

Recent evidence on pancreatic, breast, and other cancers has implicated CAFs in the metabolic reprogramming of tumor cells through dysregulation of tumor glucose, amino acid, and lipid metabolic pathways 14, 15. The metabolism of tumors is characterized by alterations in metabolic pathways or the direct acquisition of intermediate metabolites from the microenvironment to meet the increased nutritional and biosynthetic demands of rapid growth 16. The secretion of regulatory proteins by CAFs contributes to this alternative source of carbon. Alterations in protein expression can also lead to increased release of nutrient-rich material, as seen when caveolin-1 levels are reduced, leading to increased autophagy and catabolism in CAFs, releasing lactate, ketone bodies, and glutamine to create a nutrient-rich microenvironment that metabolically supports tumor growth 17. Metabolic reprogramming sustains biosynthetic demands while directly inducing metastatic pathways through substrate-mediated signaling. Indeed, CAFs have been shown to enhance glycolysis in malignant cells through FAK signaling 18, whereas serine metabolism modulates redox homeostasis to potentiate invasive capacity 19. Huang *et al.* performed single-cell transcriptomic sequencing of metastatic OS cells, observing marked upregulation of lysyl oxidase in CAFs that promoted metastasis 20. Nevertheless, despite several recent studies suggesting a relationship between abnormal levels of metabolic enzymes in CAFs and OS metastasis, the effects of CAFs on OS cell metabolism have not been elucidated and further studies are needed.

This study used CAF-like cells obtained by exposure of normal fibroblasts to conditioned medium of OS cells to define their role in OS metastasis. Non-targeted metabolomics using liquid chromatography-mass spectrometry (LC-MS) was undertaken to evaluate changes in metabolic pathways and metabolic enzymes aiming to identify key factors in CAF-like cells and the mechanisms by which they influence OS metastasis.

## Methods

### Cell culture

The human embryonic lung fibroblast cell lines WI-38 and HELF were acquired from Procell (Wuhan, China), and were authenticated by STR profiling. WI-38 and HELF cells from passages 2 to 6 following purchase were used for experiments. The human OS cell lines HOS, 143B, and GFP-tagged 143B were purchased from the ATCC (USA). HELF cells were cultured in DMEM (Gibco, 10566016) while other cells were cultured in MEM (HyClone, SH30024.01), with 10% FBS (Biological Industries, C04001-500) and 1% penicillin/streptomycin (Biosharp, BL505A) at 37 ℃ and 5% CO_2_ in a humidified incubator.

### CAF activation via external stimulation

The normal fibroblast (NF) lines WI-38 and HELF were exposed to conditioned medium (CM) from HOS and 143B OS cells, inducing activation into CAF-like cells, as described 21, 22. To generate CM, HOS and 143B cells were inoculated in 100 mm dishes and grown in MEM with FBS and antibiotics, as described above. At 60% confluence, the culture media were replaced with 10 mL of serum-free medium and cultured for a further 36 h to enable the release of cytokines and other substances, after which the FBS-free CM was collected for immediate use or storage at -80 ℃.

CAF activation via external stimulation was performed using 60% confluent WI-38 and HELF cells in 60 mm dishes. The HOS/143B-CM was filtered through a sterile 0.22 µm filter (Biosharp, BS-PES-22) to remove cellular debris and impurities, after which 10% FBS was added to the filtrate, 5 mL of which was used to replace the medium of NFs. Culture was continued for 48 h to induce the activation of NFs to CAF-like cells. Flowchart of this procedure is provided in Supplementary Figure 1A.

CM of NFs or CAF-like cells was also prepared using the above method, followed by the addition of 10% FBS when used for OS cell co-cultures (Supplementary Figure 1B).

### Isolation and culture of primary CAFs and NFs

OS tissues and matched normal skeletal muscle tissues were finely cut into ~1mm^3^ pieces after three rinses of saline and then digested with 0.15% IV type collagenase (Sigma Aldrich, C5138-100MG) for 2 h at 37°C. The digested tissue fluid was filtered with a 70 μm filter and centrifuged at 1400 rpm for 10 min, then cell precipitation was collected and seeded into T25 culture flask. The primary CAFs and NFs were washed with 10% penicillin/streptomycin and incubated in M199 (Hyclone, SH30253.01) and DMEM (Gibco, 10566016) at 1:2 volumetric ratio, supplemented with 10% FBS and 1% penicillin/streptomycin at 37 ℃ and 5% CO_2_.

### qPCR

Total RNA was extracted from OS cells or fibroblasts using TRIzol (T9424, Sigma) with concentrations and purity measured with a NanoDrop 2000 spectrophotometer (Thermo Fisher), and reverse-transcription to cDNA using HiScript III RT SuperMix (+gDNA wiper) (Vazyme, R323). Amplification with qPCR was conducted with ChamQ SYBR qPCR Master Mix (Vazyme, Q311), according to the provided directions, on a StepOnePlus Real-Time PCR System (Applied Biosystems, GeneAmp 9700). Primers were synthesized by GeneCreate Biotech (Wuhan, China) and sequences are provided in Supplementary Table 1. GAPDH was utilized for data normalization.

### Western blotting

Cells were lysed with RIPA buffer (Beyotime, P0013) with 1% PMSF (Beyotime, ST505) and a protease and phosphatase inhibitor cocktail (Beyotime, P1045). Proteins were resolved on SDS-PAGE and electroblotted to PVDF membranes. The blots were blocked (5% BSA, 1 h, room temperature) and treated with primary antibodies (overnight, 4 ℃), followed by incubation with appropriate HRP-conjugated secondary antibodies (Servicebio, GB23301/GB23303). Proteins were ultimately visualized using an ultra-sensitive ECL luminescence reagent (Bio-Rad, 1705061) with an infrared imaging system (UPV ChemiDoc-It 510, Thermo Fisher Scientific).

Antibodies against GAPDH, fibronectin, Smad2, E-cadherin, N-cadherin, Vimentin, PSAT1, mTOR, and p-mTOR (Ser2448) were purchased from Proteintech (60004-1-Ig, 15613-1-AP, 12570-1-AP, 20874-1-AP, 22018-1-AP, 10366-1-AP, 67619-1-Ig, 28273-1-AP, and 67778-1-Ig, respectively), α-SMA from Abcam (ab7817), β-actin, STAT3, and p-PI3K (P85α/γ/β-Y467/Y199/Y464) from Abclonal (AC006, A1192, AP0854, respectively), FAP, IL-6, p-STAT3 (Ser727), and p-S6K (Thr389/Thr412) from Affinity (AF0739, DF6087, AF3294, AF3228, respectively), TGF-β and p-Smad2 (Ser465) from Bioss (bs-0086R, bs-2224R, respectively), and TGFBR and PI3K from CST (5544, 4292, respectively).

### Immunofluorescence staining

WI-38 and CAF-like cells were allowed to adhere on 20 mm-diameter coverslips for 12 h, followed by fixation (4% paraformaldehyde, 30 min), and permeabilization (0.5% Triton X-100 [Beyotime, P0096], 15 min). After blocking with 5% BSA, the cells were stained overnight with the primary antibody against α-SMA (Abclonal, A17910) at 4 ℃, followed by treatment with a Cy3-conjugated secondary antibody (Abclonal, AS007) for 1 h away from light and nuclear staining with Hoechst (Beyotime, C1017) for 30 min. Immunofluorescence was assessed and imaged with a fluorescence microscope (Olympus, BX53).

After fixation by the above method, HOS and 143B cells were subjected to dual immunofluorescence co-staining through sequential incubations. Initially, the cells were probed with the first primary antibody targeting PSAT1 (Proteintech, 67619-1-Ig) followed by its corresponding Cy3-labeled secondary antibody. Subsequently, the samples were incubated with the second primary antibody against mTOR (Proteintech, 28273-1-AP) in combination with its Cy5-conjugated secondary antibody.

### CCK-8 assays

Cell viability and time-dependent proliferation were assessed by CCK-8 assays (Biosharp, BS350B). WI-38, HELF, or HOS cells (2000/well) were inoculated in 96-well plates and grown for 24 h, after which the media were discarded and the cells were treated with CM or MEM with 10% FBS for 24, 48, or 72 h under normal growth conditions, followed by addition of 100 μL of 10% CCK-8 reagent for 2 h. Absorbances at 450 nm were read using a microplate reader (PerkinElmer, Enspire).

### Clonogenic survival assay

HOS cells (100/well) were grown in 6-well plates for 24 h under 37 ℃, after which the media were replaced with CAF-CM, WI-38-CM, or MEM containing 10% FBS and culture continued for 48 h. After that, CM was replaced with normal medium, and cells were further allowed to grow for 10 days to form clusters. The colonies were then fixed (4% paraformaldehyde) and stained (0.1% crystal violet, 30 min) and those with a minimum of 50 cells were counted.

### Wound-healing assay

Cells were inoculated in 6-well plates (each well containing 2 mL MEM with 10% FBS) and grown until 90% confluent. Scratches were then made with a 200 μL pipette tip, followed by gentle rinsing with PBS. The cells were then treated with 2 mL of CM or MEM with no FBS and grown for 36 h, after which the cells were imaged microscopically at the 0, 12, 24, or 36 h time points.

### Transwell assay

HOS or 143B cells (2×10^4^ or 3×10^4^/well) were inoculated in the upper compartment of a Transwell insert and treated with 200 μL of specific treatments without FBS (CAF-CM, WI-38-CM, or MEM). The lower compartment contained MEM with 10% FBS. The cells were grown for 36 h and then fixed and stained as above. Cells in the upper compartment that had not migrated were gently removed, while the migrated cells were allowed to dry and were imaged microscopically.

### Assays for tumor lung metastasis* in vivo*

90% of metastatic OS occurs in the lungs 4, so we used the lung metastasis model for *in vivo* experiments. BALB/c nude mice (female, 6 weeks old) were purchased from the Hubei Provincial Center for Disease Control and Prevention (HBCDC). To verify the metastasis-promoting effect of CAF-like cells *in vivo*, 10 mice were randomly allocated to two groups (N1 = 5), and 2×10^6^ 143B cells with or without an equal number of CAF-like cells in 100 μL PBS were injected into the tail veins of the mice. To investigate the effects of PSAT1 and mTOR inhibitor *in vivo*, 15 animals were randomly allocated to three groups (N2 = 5). Group 1 received 2×10^6^ GFP-tagged 143B cells mixed with an equal number of CAF-like cells (also injected into the tail vein), whereas the other two groups received 2×10^6^ GFP-tagged 143B cells overexpressing PSAT1 mixed with CAF-like cells. After 7 days, animals in the first and second groups were given intraperitoneal injections of PBS three times per week, while animals in the third group received the mTOR inhibitor rapamycin (2 mg/kg per mouse 23) three times a week for four weeks. Bioluminescence imaging was conducted with an IVIS Lumina K Series III, with normalization of the image radiance using Living Image (PerkinElmer, USA). Mice were euthanized after six weeks and lung tissues were harvested, with paraffin-embedded lung tissue sections (4 µm) stained by hematoxylin and eosin (H&E) for histological assessment.

### Metabolomics analysis

HOS cells were treated with CAF-CM or WI-38-CM (10% FBS) for 36 h, rinsed with PBS, scraped, and centrifuged. Cell pellets (200 mg/sample) were flash-frozen in liquid nitrogen and stored at -80 ℃. Metabolomics analysis was carried out by Anachro Technologies Inc. Co., Ltd. using non-targeted LC-MS. Metabolites were extracted with methanol, dried under nitrogen, reconstituted in acetonitrile (v/v, 1:1), and separated on an ACQUITY UPLC HSS T3 C18 column (100*2.1 mm 1.8 μm; Waters) using a gradient of 0.1% formic acid (A) and methanol (B) at 0.3 mL/min. ESI parameters included capillary voltages of 3.8 kV (positive ion mode) and 3.2 kV (negative ion mode), sheath gas flow (40 Arb, 300 ℃).

Data were processed via MS-DIAL for peak alignment and metabolite annotation (MSBank database). Unsupervised principal component analysis (PCA) and supervised partial least squares discriminant analysis (PLS-DA) were conducted to distinguish metabolic profiles, with significance defined as *P* < 0.05, *VIP* ≥ 1, and *FC* ≥ 2. The KEGG database (http://www.genome.jp/kegg/) and QEAmodel of MSEA (https://www.metaboanalyst.ca/) were used for analysis of metabolic pathways using the SMPDB database (https://smpdb.ca) to calculate the enrichment (FE) and* P*-values.

### Bioinformatics analysis

As described in our previous study 24, the microarray dataset GSE19276_GPL6848 was downloaded from the GEO database (https://www.ncbi.nlm.nih.gov/geo/); this includes 44 OS and 5 normal tissue samples, and was used for comparing PSAT1 expression in OS. The transcriptomic and clinical data of 85 OS cases including survival information were downloaded from UCSC Xena (https://xenabrowser.net/datapages/) for prognostic analysis. The optimal cutoff PSAT1 level was determined using X-tile software (Yale University, USA) and cases were allocated to high- and low-expression cohorts for Kaplan-Meier analysis. GSEA in the “clusterprofiler” package in R was utilized to examine the biological pathways associated with PSAT1 in pan-cancer.

### Tissue immunohistochemistry and immunofluorescence analysis

Primary tumor tissues obtained by surgical resection from 10 OS cases were immediately frozen in liquid nitrogen and stored at -80 ℃ for further investigation of PSAT1 levels using qPCR, western blotting, and immunohistochemistry.

The paraffin-embedded samples were sectioned (4 μm) and deparaffinized. Following antigen retrieval and blocking, the sections were incubated with the primary anti-PSAT1 antibody (Proteintech, 10501-1-AP) overnight at 4 ℃, and then with the secondary antibody for 1 h. The sections were stained with DAB and counterstained with hematoxylin.

Sections of seven OS lung metastases were used for immunofluorescence analysis. These were treated as above and then incubated in succession with the primary antibody anti-PSAT1 (Proteintech, 10501-1-AP), HRP-labeled secondary antibody (Servicebio, GB23303), FITC-Tyramide (TSA) dye (Servicebio, G1222), primary antibody against α-SMA (Abclonal, A17910), Cy3-labeled secondary antibody (Servicebio, GB21303), and Hoechst nuclear stain (Beyotime, C1017), according to the provided directions, before imaging.

### Multiplex Cytometric Bead Array (CBA) and ELISA

The HOS-CM, CAF-CM, and WI-38-CM (all without FBS) were collected for multiplex cytokine analysis. Cytokines (GM-CSF, IFN-α, IFN-γ, IL-1β, IL-10, IL-12p70, IL-13, IL-17A, IL-2, IL-4, IL-5, IL-6, IL-8, TNF-α) were quantified using the magEasyQPlex human 14-plex Flow Assay Kit (PLEM100, Laizee Biotech) following the manufacturer's instructions. TGF-β was measured by the TGF-β ELISA KIT (LER822-1, Laizee Biotech) after acidizing and diluting 1.5 times under the guidance of instruction, and the percentage of TGF-β released was calculated.

### Cycloheximide (CHX) chase assay

CHX chase assay was performed to investigate post-translational degradation dynamics. Following 24 h transfection with si-PSAT1 or si-NC, cells were treated with 10% FBS MEM medium containing 100 μg/mL CHX (AbMore, M4879) under standard culture conditions (37 ℃, 5% CO₂). Cells were harvested at 0, 4, 12, and 24 hours post-CHX treatment and lysed for western blotting to evaluate temporal changes in mTOR protein levels.

### Statistical analysis

Data were analyzed using R (version 4.1.1, Austria), Image J 1.52 2 (NIH, USA), and GraphPad Prism 7.0.0 (GraphPad Software, USA). Data are presented as mean ± SD. Two-tailed unpaired t-tests were used for comparisons between two groups, and one-way ANOVA was used for comparisons of multiple groups. Kaplan-Meier curves were compared using log-rank tests. Wilcoxon tests were utilized to compare the levels of PSAT1 between paired tumors and normal tissues. Correlation analysis between PSAT1 and α-SMA was performed using the nonparametric Spearman's analysis. *P* < 0.05 was considered to be statistically significant. *P < 0.05, **P < 0.01, ***P < 0.001.

### Ethics statement

Animal experiments received approval from the IACUC at Hubei Provincial Center for Disease Control and Prevention, while human studies were approved by the Medical Ethics Committee at Union Hospital, Tongji Medical College, Huazhong University of Science and Technology. All participants provided written informed consent.

## Results

### HOS and 143B activated WI-38 and HELF *in vitro* to become CAFs-like cells

**Figure 1:**
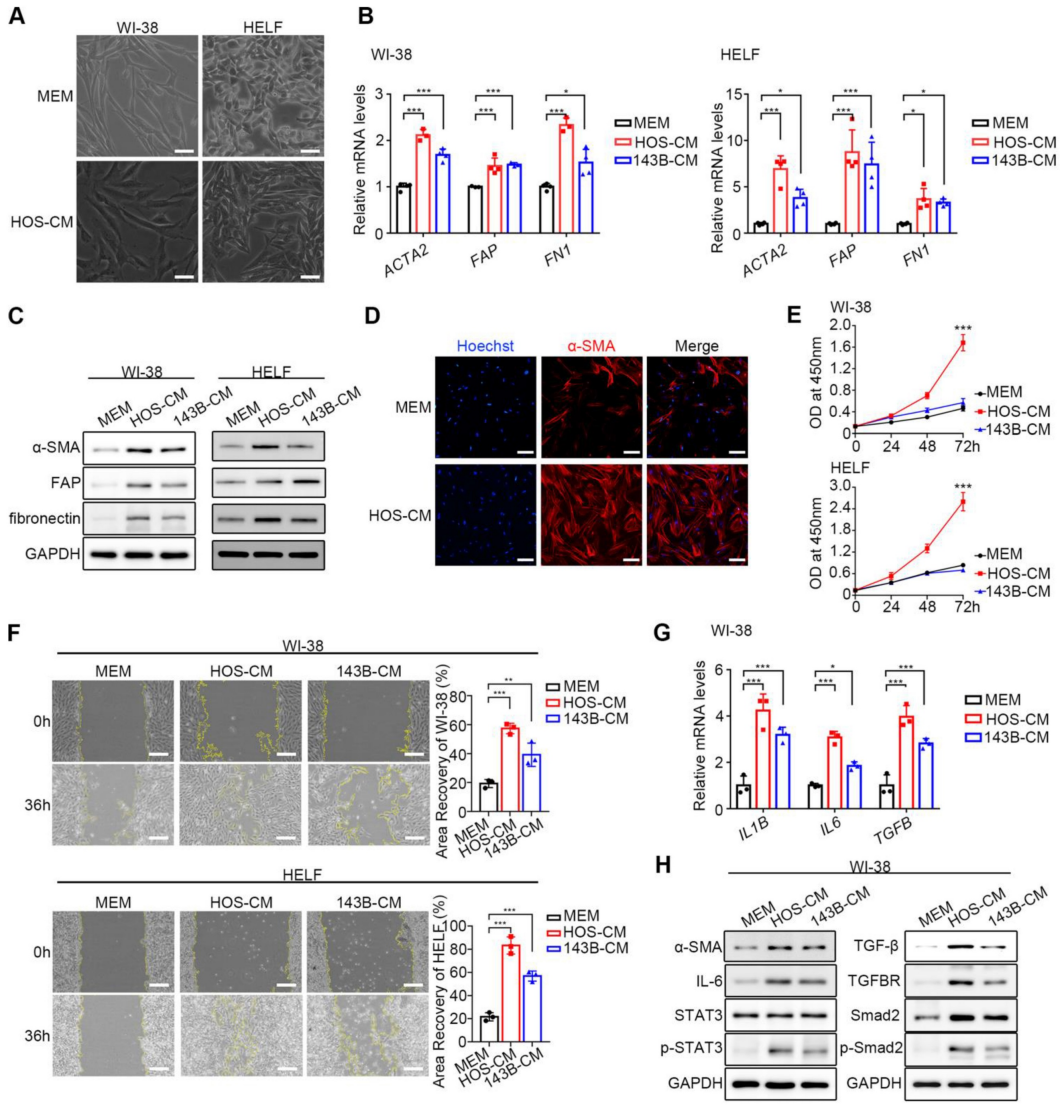
Obtaining of CAF-like cells. (A) The morphological images of NFs and CAF-like cells, scale bars, 50 µm. CAF-like cells were treated with HOS-CM, while NFs were cultured in MEM. (B) Relative mRNA expression of CAF biomarkers in WI-38 and HELF cells by q-PCR, the indicated results represent the mean ± SD (n=4), * *P* < 0.05, ** *P* < 0.01, *** *P* < 0.001. WI-38 and HELF cells were treated with HOS-CM and 143B-CM, while the control group was the MEM group. (C) The protein level of CAF biomarkers in WI-38 and HELF cells by western blotting analysis. (D) The expression of α-SMA in normal WI-38 and CAF-like cells were assayed by immunofluorescence staining, scale bars, 100 µm. (E) Proliferation ability of NFs and CAF-like cells by CCK-8 assay was displayed as OD value at 450 nm. (F) Migration ability of NFs and CAF-like cells were analyzed by wound-healing assays and expressed as area recovery rate, scale bar, 200 µm. (G) Relative mRNA expression of inflammatory factors in WI-38 by q-PCR, the indicated results represent the mean ± SD (n=3), * *P* < 0.05, ** *P* < 0.01, *** *P* < 0.001. (H) The protein level of IL-6/STAT3 and TGF-β receptor pathways in WI-38 by western blot analysis.

HOS/143B-CM was used for the co-culture of WI-38 and HELF fibroblasts, while MEM was used for control. After co-culture with HOS/143B-CM, fibroblasts exhibited a spindle-shaped morphology with branched cytoplasmic extensions and enlarged nuclei (Figure 1A). To confirm the successful activation of CAF-like cells, the mRNA and protein levels of the biomarkers *ACTA2* (α-SMA),* FAP* (Fibroblast activation protein, FAP), and *FN1* (fibronectin) were examined and found to be significantly elevated (Figure 1B, Figure 1C). In addition, α-SMA fluorescence was higher in CAF-like cells than in WI-38 cells, further confirming the expression of the CAF indicators (Figure 1D). CCK-8 proliferation assay (Figure 1E) and wound-healing assay (Figure 1F) demonstrated that CAF-like cells showed significantly enhanced proliferative and migratory capacities compared to NFs. Another feature distinguishing CAFs from resting fibroblasts is the enhanced production of inflammatory factors, particularly IL-1β, IL-6, and TGF-β 9. The qPCR analysis showed that CAF-like cells in the HOS/143B-CM co-culture group transcribed* IL1B*, *IL6*, and *TGFB* at higher levels compared to those in the WI-38 controls (Figure 1G). Furthermore, previous studies have suggested that the activation of resting fibroblasts into CAF-like cells is largely dependent on the IL-6/STAT3 and TGF-β receptor axes 9, 21, 22. Here, the expression and phosphorylation levels of key proteins in the IL-6/STAT3 and TGF-β receptor pathways were examined, finding that in addition to the elevation in α-SMA, the cellular IL-6 levels and STAT3 phosphorylation levels were also elevated in the co-culture group, as were the levels of TGF-β and its receptor TGFBR, as well as Smad2 and Smad2 phosphorylation (Figure 1H), suggesting activation of the IL-6/STAT3 and TGF-β/Smad2 axes. These findings indicate the successful production of CAF-like cells.

To further investigate how OS cells activate CAF-like cells, we analyzed the secretory profiles of HOS-CM and normal NF-CM using a 14-plex CBA and ELISA. Elevated levels of GM-CSF, IFN-α, IFN-γ, IL-6, IL-8, and TGF-β were identified in HOS-CM compared to NF-CM (Supplementary Figure 2A-C). Treatment of WI-38 cells with the TGF-β specific inhibitor SB431542 (10 μM, Sigma) significantly reduced the expression of the activation marker FAP (Supplementary Figure 2D), suggesting a critical role of TGF-β in OS-driven CAF activation.

### CAF-like cells promote OS cell migration

As described in Supplementary Figure 1B, CAF-CM was used for co-culture with OS cells, together with two controls, namely, WI-38-CM and MEM. Considering that tumor migration not only reflects cell motility but also correlates with cellular activity, CCK-8 and clonogenic survival assays were first performed to investigate the effect of CAF-CM co-culture on the growth of HOS cells. No significant differences in the CCK-8 assay results of the three HOS cell groups at 24, 48, and 72 h were found (all *P* > 0.05) (Supplementary Figure 3A). The clonogenic assay results indicated that approximately 70 clones were formed in all three groups on day 10 (all *P* > 0.05) (Supplementary Figure 3B). These findings suggest that CAF-like cells did not influence the survival of OS cells.

**Figure 2:**
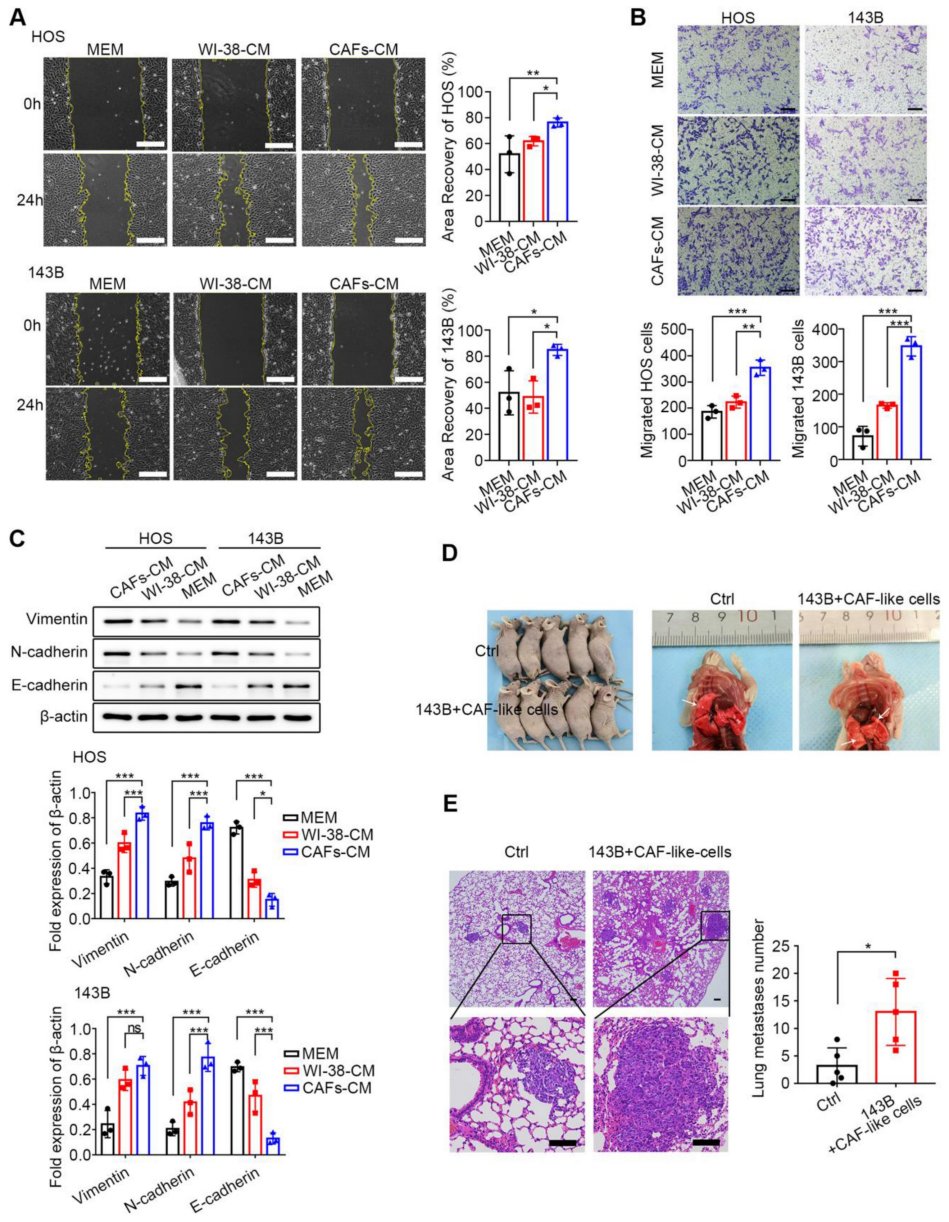
CAF-like cells enhance HOS and 143B migration ability. (A) Migration of HOS and 143B after CAFs-CM treatment was analyzed by wound-healing assay and expressed as area recovery rate (scale bars, 200 µm), the indicated results represent the mean ± SD (n=3), * *P* < 0.05, ** *P* < 0.01, *** P < 0.001. (B) Cell migration ability in Transwell assay was displayed as migrated cell number (scale bars, 100 µm), the indicated results represent the mean ± SD (n=3), * *P* < 0.05, ** *P* < 0.01, *** *P* < 0.001. (C) Protein expression of E-cadherin, N-cadherin and Vimentin in HOS and 143B after CAFs-CM treatment, the indicated results represent the mean ± SD (n=3), * *P* < 0.05, ** *P* < 0.01, *** *P* < 0.001. (D) The tumor metastasis node of lungs in different groups. (E) Representative images of histological changes in mouse lungs evaluated by H&E staining, scale bars, 200 µm. Student's t test analysis for comparisons between two groups of lung metastases number, represented by the mean ± SD (n=5), * *P* < 0.05, ** *P* < 0.01, *** *P* < 0.001.

The wound-healing and Transwell assays were undertaken to assess the influence of CAF-like cells on the migration of HOS and 143B cells. In the wound-healing assays, after 24 h, it was found that co-culture with CAF-CM enhanced the recovery of the wounded area in both HOS and 143B cells relative to the control groups (*P* < 0.05) (Figure 2A). In the Transwell assays, the number of cells in the upper compartment was 2*10^4^ cells. After 36 h, there was a marked increase in the number of migrated cells in the CAF-CM group relative to the controls (*P* < 0.05) (Figure 2B). These findings indicate that CAF-like cells promoted the migration of HOS and 143B cells. The EMT is a critical process involved in tumor cell migration and is associated with inhibition of E-cadherin and raised levels of both N-cadherin and Vimentin, which are thus markers of the EMT. Here, it was found that CAF-CM co-cultured HOS and 143B cells expressed lower levels of E-cadherin but increased N-cadherin and Vimentin, suggesting that the CAF-like cells promoted the EMT in both HOS and 143B cells (Figure 2C).

Since 90% of OS metastases occur in the lungs, the effects of CAF-like cells on 143B cell metastasis were examined in a mouse model. A total of 2×10^6^ 143B cells with or without an equal number of CAF-like cells were administered intravenously, and lung tissues were collected after six weeks. As expected, the lungs in the CAF co-culture group contained greater numbers of metastatic nodules than the controls (*P* < 0.05) (Figure 2D). Further H&E staining indicated that mice receiving both CAF-like and 143B cells had more lung metastases relative to the controls (Figure 2E).

To further characterize the secretory profiles of WI-38-CM and CAF-CM, we employed a 14-plex CBA and ELISA. Analysis revealed a marked change in pro-metastatic cytokines, including GM-CSF, IL-1β, IL-6, IL-8, and TGF-β, in CAF-CM compared to WI-38-CM (Supplementary Figure 4A-C).

### CAF-like cells promote reprogramming of the serine metabolic pathway in HOS cells

While previous studies have reported that CAFs affect tumor metabolic reprogramming to promote metastasis, there is limited knowledge on the effects of CAFs on the metabolic profiles of OS cells. To evaluate changes in OS metabolism induced by CAF-CM and their effects on OS migration, the metabolic profiles of OS cells were examined using LC-MS non-targeted wide-screen metabolomics.

**Figure 3:**
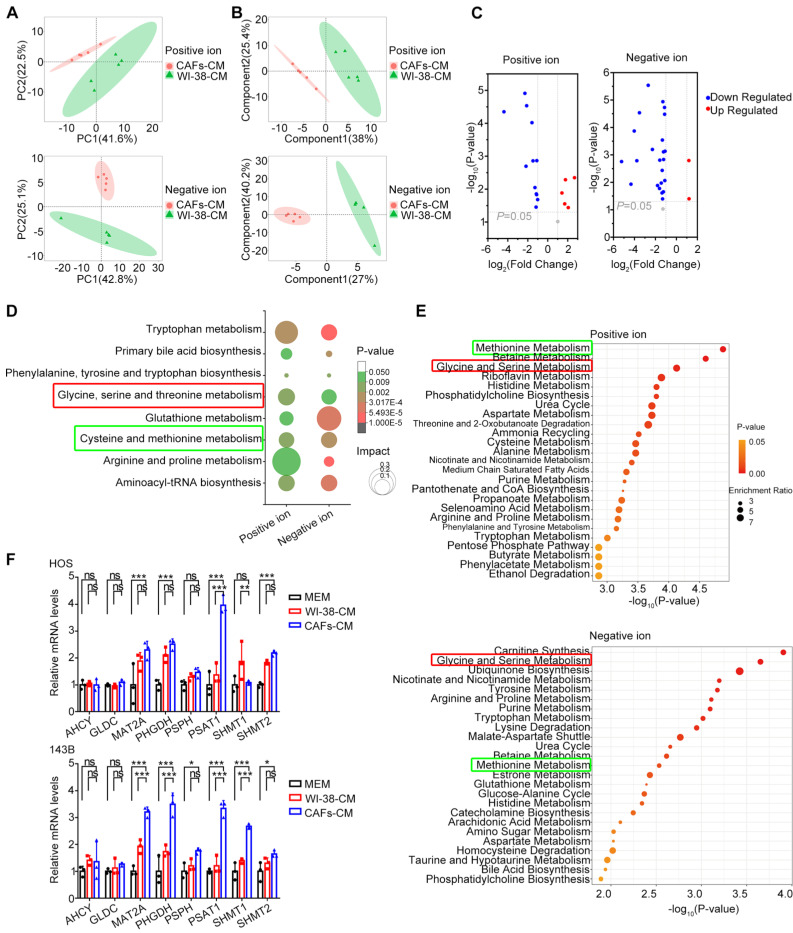
CAF-like cells reprogram OS cell metabolism. (A) PCA of metabolite profiles in HOS co-culturing with CAFs-CM and WI-38-CM. (B) PLS-DA of metabolite profiles in HOS. (C) Volcano plot of differential metabolites in HOS co-cultured with CAFs-CM and WI-38-CM. (D) Bubble plots of major KEGG metabolic pathways in HOS co-culturing with CAFs-CM. (E) Top 25 of MSEA-enriched metabolite sets in HOS co-culturing with CAFs-CM. (F) Relative mRNA level of key enzyme-related genes in methionine metabolism and serine metabolism by q-PCR, the indicated results represent the mean ± SD (n=3), * *P* < 0.05, ** *P* < 0.01, *** P < 0.001.

The acquired data were evaluated with the MSBank database. In the positive-ion mode, the number of primary pairs of compounds was 6229 with 160 secondary compound pairs, while in the negative-ion mode, 5079 primary pairs were identified with 140 secondary pairs; ultimately, 300 metabolites were identified by matches in MSBank. Both PCA (Figure 3A) and PLS-DA (Figure 3B) were utilized to visualize differences in metabolites and observe the effects of CAF-CM co-culture on the metabolic profiles of HOS cells. In both positive and negative ion modes, the results indicated satisfactory clustering of samples within the same group, with high sample reproducibility; the samples in the two groups were completely separated, indicating significant metabolic differences between the two groups of HOS cells. The volcano plot shows the differential metabolites that satisfied the criteria of t-test *P* < 0.05,* VIP*≥1, and *FC*≥2 (Figure 3C). A total of 42 differential metabolites were identified, of which 5 were up-regulated and 12 down-regulated in the positive ion mode, while in the negative ion model, 2 were up-regulated and 23 down-regulated.

KEGG enrichment analysis of metabolic pathways was performed to determine which specific metabolic pathways were affected by CAF-like cells. In the positive ion mode, 300 metabolites were found to be associated with 39 KEGG pathways in HOS cells treated with CAF-CM, relative to the controls, of which 24 differed significantly (*P* < 0.05) (Supplementary Table 2). In the negative ion mode, CAF-CM influenced 52 KEGG pathways in HOS cells, with 23 showing significance (*P* < 0.05) (Supplementary Table 3). The KEGG results are illustrated as bubble plots (Figure 3D), and eight metabolic pathways were found to be significant in both the positive ion and negative ion modes (*P* < 0.05), most of which were associated with the metabolism of various amino acids. Alterations in amino acid metabolic pathways may thus contribute to the influence of CAF-like cells on OS migration.

MSEA analysis was then utilized to assess the enrichment of metabolites that showed little difference between the groups, and metabolite sets were ranked according to fold enrichment and the significance of differences. The results showed that methionine metabolism, as well as glycine and serine metabolism, ranked among the top 25 metabolite sets in both the positive and negative ion modes (Figure 3E). Differences in the serine metabolic pathway were likewise found to be significant in both positive and negative ion mode MSEA results. The relative abundance of metabolites involved in methionine and serine metabolism is shown in Supplementary Figure 5.

These results indicate the key nature of the methionine and serine metabolic pathways in OS. Therefore, the levels of key genes in these pathways were verified by qPCR. This showed that *PSAT1* was significantly up-regulated in HOS cells co-cultured with CAF-CM (*P* < 0.05 vs. both controls) (Figure 3F). In addition, the expression of methionine adenosyltransferase 2A (MAT2A), 3-phosphoglycerate dehydrogenase (PHGDH), PSAT1, and serine hydroxymethyl transferase 1 (SHMT1) was markedly higher in CAF-CM-co-cultured 143B cells (*P* < 0.05 for both controls) (Figure 3F). These results demonstrate up-regulated expression of PSAT1, a key enzyme in serine metabolism, in OS cells by CAF-like cells.

### CAF-like cells upregulate the serine metabolism enzyme PSAT1 in OS

**Figure 4:**
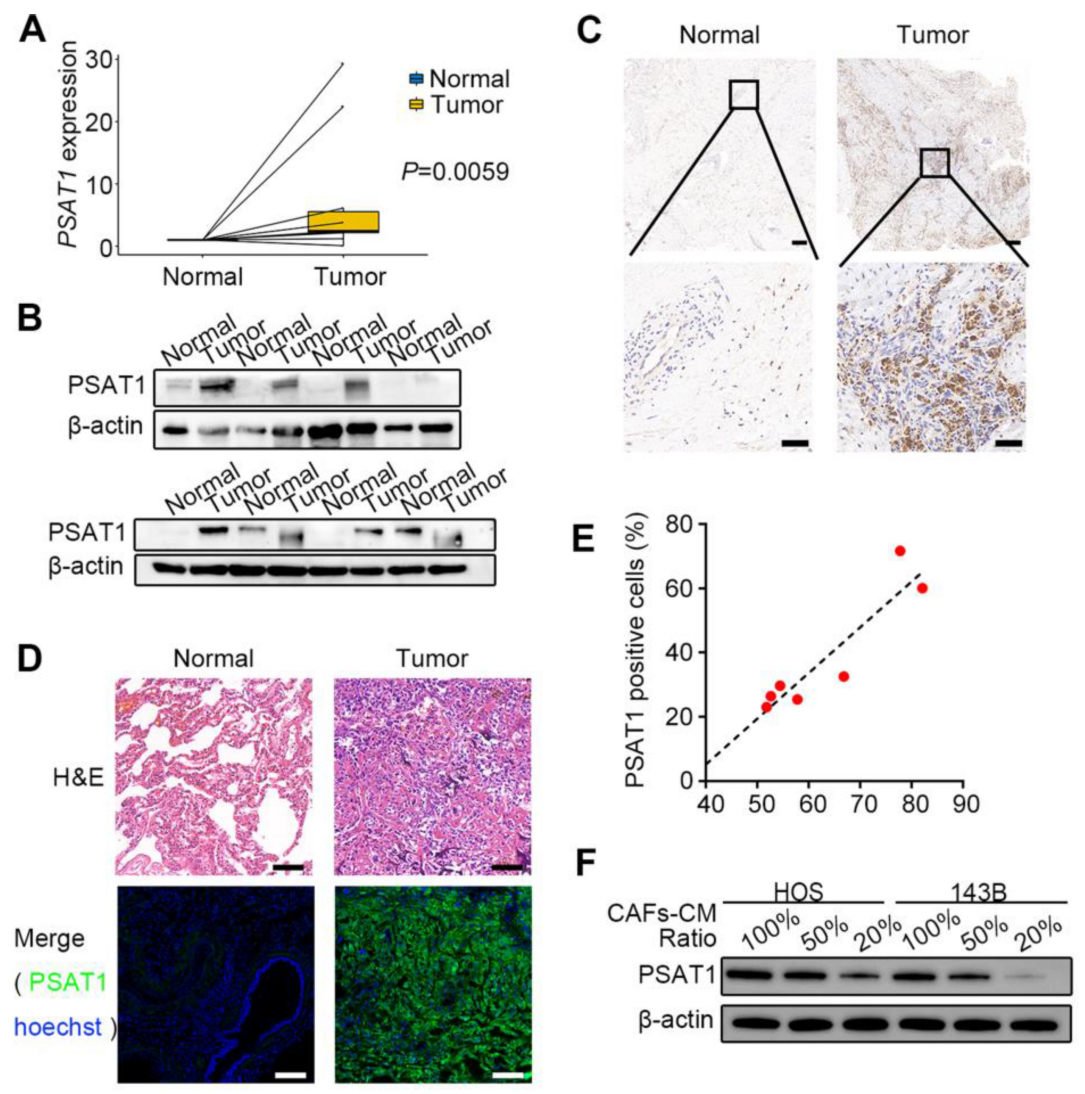
PSAT1 expression is increased in OS and positively correlates with CAFs. (A) Relative mRNA level of PSAT1 in 10 pairs of OS primary tumors and normal tissues by q-PCR, *P*=0.0059 24. (B) Protein levels of PSAT1 in OS primary tumors and paired normal tissues by western blotting analysis 24. (C) Representative images of immunohistochemistry staining for PSAT1 in OS primary tumors and paired normal tissues, scale bars, 200 µm (up), 50 µm (down) 24. (D) Representative images of H&E staining and PSAT1 immunofluorescence staining in OS lung metastases and paired normal lungs, scale bars, 50 µm. (E) Scatter plot of PSAT1 and α-SMA expression levels in 7 cases of OS lung metastases, Spearman's correlation coefficient=0.857, *P*=0.014. (F) The changes of PSAT1 protein levels under different CAFs-CM ratios in HOS and 143B.

Public databases were first used to analyze PSAT1 expression levels in OS tissues. Transcriptomic data from the GEO dataset (GSE19276_GPL6848) suggested that PSAT1 levels were raised in OS tissues relative to normal tissues (log*FC*=1.618, adjusted* P*=0.041); the heatmap (Supplementary Figure 6A) and volcano plot (Supplementary Figure 6B) are shown in the supplementary materials. Further survival analysis was performed using the GDC Target-OS dataset with clinicopathological characteristics shown in Supplementary Table 4. An optimal cutoff value of 4.8 calculated by X-tile was used to divide 85 OS cases into high-expression (≥4.8, n=38) and low-expression (<4.8, n=47) groups, with Kaplan-Meier analysis showing markedly lower survival rates in the high-expression group (*P*=0.0178) (Supplementary Figure 6C). We further analyzed *PSAT1* expression in metastatic versus non-metastatic OS patients. The results showed higher *PSAT1* levels in metastatic cases, though the difference did not reach statistical significance (*P* > 0.05) (Supplementary Figure 6D). Next, PSAT1 levels were assessed in OS surgical samples. The basic clinical information of the 10 cases with resected primary lesions is summarized in Supplementary Table 5. The qPCR results showed marked up-regulation of PSAT1 transcription in these samples relative to normal control tissues (*P*=0.0059) (Figure 4A). Western blotting (Figure 4B) and immunohistochemical staining of paraffin sections (Figure 4C) confirmed that PSAT1 protein levels were higher in OS primary tissues relative to the controls. The clinical data of seven patients with resected lung metastases are shown in Supplementary Table 6. Immunofluorescence staining for PSAT1 and α-SMA was performed on these lesions together with paired normal lung tissues (Figure 4D, Supplementary Figure 7A-B). The positive cell ratio and positive cell density of PSAT1 in the seven pairs of samples are shown in Supplementary Table 7, indicating marked elevation in PSAT1 in tumor tissues (all *P* < 0.05). The immunofluorescence results indicated that PSAT1 fluorescence in the metastatic tissues was consistent with the H&E-stained tumor areas, demonstrating high levels of PSAT1 in OS lung metastases. A positive cell ratio of PSAT1 to α-SMA was further calculated, with the scatter plot showing a positive association between the expression levels of PSAT1 and α-SMA (Figure 4E) (*P* < 0.05). The above results link CAFs with elevated PSAT1 expression in both primary and metastatic OS.

We next assessed PSAT1 protein levels in HOS and 143B cells co-cultured with CAF-CM at varying ratios. Western blotting revealed a dose-dependent upregulation of PSAT1 levels, correlating with increasing proportions of CAF-CM (Figure 4F). We isolated primary CAFs and NFs from OS patient issues (Supplementary Figure 8A-B), and further confirmed that CAF-CM co-culture significantly upregulated PSAT1 protein expression in OS cells (Supplementary Figure 8C).

### PSAT1 knockdown reduces HOS and 143B cell migration

**Figure 5:**
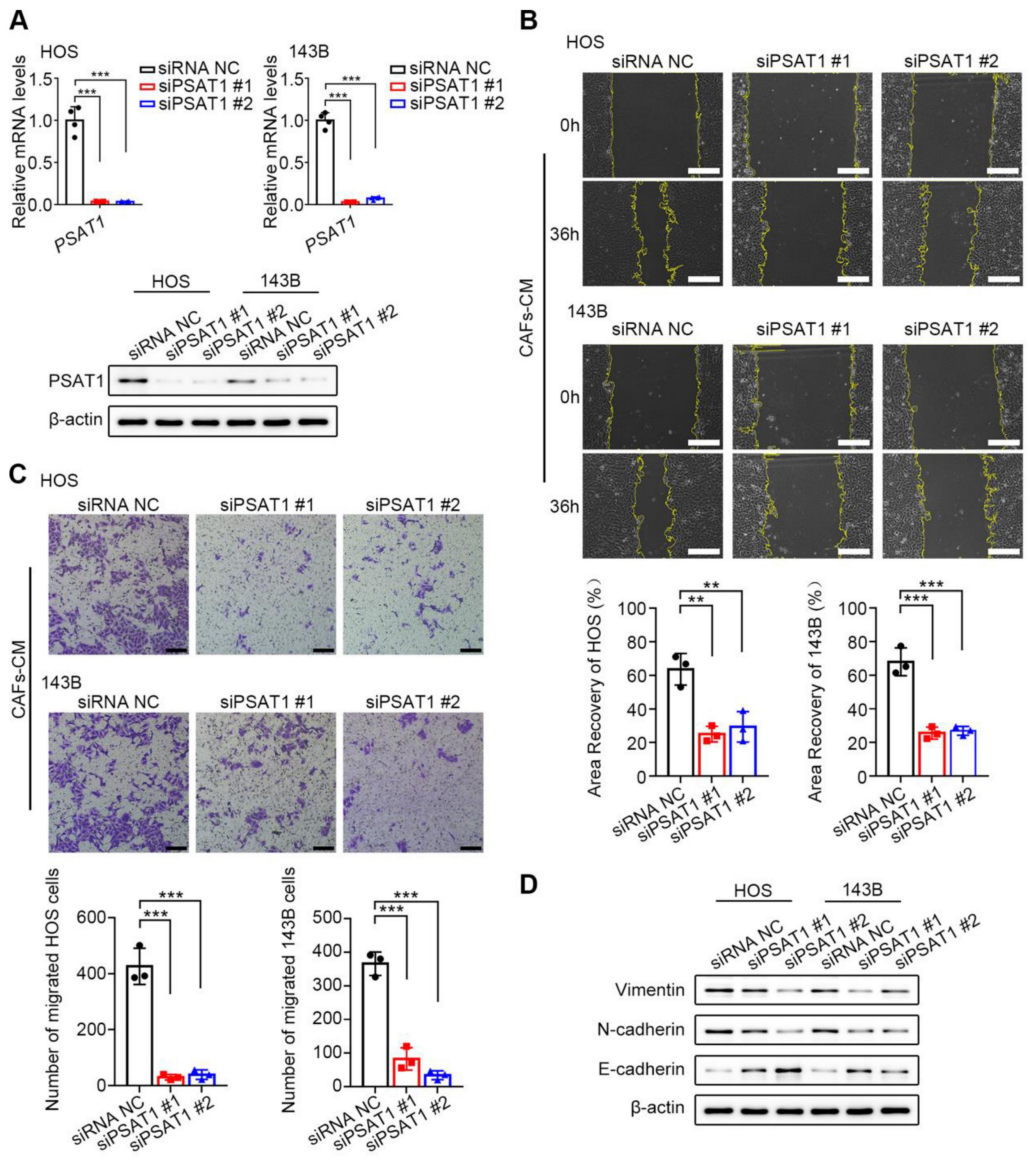
CAFs promote OS migration through enhanced PSAT1. (A) siPSAT1 interference in HOS and 143B cells by qPCR and western Blotting. (B) siPSAT1 inhibits migration of HOS and 143B after CAFs-CM treatment, analyzed by wound-healing assay (scale bars, 200 µm), the indicated results represent the mean ± SD (n=3), * *P* < 0.05, ** *P* < 0.01, *** P < 0.001. (C) siPSAT1 inhibits cell migration ability of HOS and 143B after CAFs-CM treatment, analyzed by Transwell assay (scale bars, 100 µm), the indicated results represent the mean ± SD (n=3), * *P* < 0.05, ** *P* < 0.01, *** *P* < 0.001. (D) Protein expression of E-cadherin, N-cadherin and Vimentin in HOS and 143B after siPSAT1.

To assess the functions of PSAT1 in OS cell migration, PSAT1 expression was knocked down in HOS and 143B cells by siRNA (Figure 5A). Approximately 6 h after siRNA transfection, the culture media were replaced with CAF-CM containing 10 % FBS. Subsequently, wound-healing (Figure 5B) and Transwell (Figure 5C) assays were performed to evaluate cell migration. Wound healing was markedly reduced in the knockdown cells relative to the controls at 36 h (all* P* < 0.05). In the Transwell assays, 3*10^4^ OS cells were seeded in serum-free CAF-CM, while the lower compartment contained MEM with 10% FBS. After 36 h of incubation, a marked reduction in the number of migrated cells was observed in the knockdown group relative to the controls (all *P* < 0.05). In addition, the ability of PSAT1 knockdown to inhibit the EMT was examined. After 6 h of siRNA transfection, the culture media were replaced with CAF-CM with 10% FBS, and the culture was continued for 36 h. Western blotting showed that the levels of both N-cadherin and Vimentin were reduced in the knockdown cells, while those of E-cadherin were enhanced, suggesting suppression of the EMT in HOS and 143B cells (Figure 5D). The above results indicated that CAF-like cells promote cell migration and the EMT in HOS and 143B cells through up-regulation of PSAT1.

### PSAT1 promotion of HOS and 143B cell migration is dependent on mTOR/S6K activation

To further investigate the potential mechanism by which PSAT1 promotes OS cell migration, it was first evaluated whether PSAT1, as a key serine synthase, might induce migration by promoting serine production. However, wound-healing assays using varying concentrations of serine supplements revealed that increased serine did not affect HOS cell migration (Supplementary Figure 9). As previous studies have reported that PSAT1 has additional non-metabolism-dependent molecular regulatory functions 25, it was hypothesized that PSAT1 may control OS cell migration by modulating molecular signals. Next, GSEA was performed to examine biological pathways associated with PSAT1 in pan-cancer. Supplementary Figure 10 shows the GSEA enrichment results of six representative cancer types, and it was found that PSAT1 was associated with several important tumor-associated pathways, including those involving E2F transcription factors, mTOR signaling, and MYC targets.

**Figure 6:**
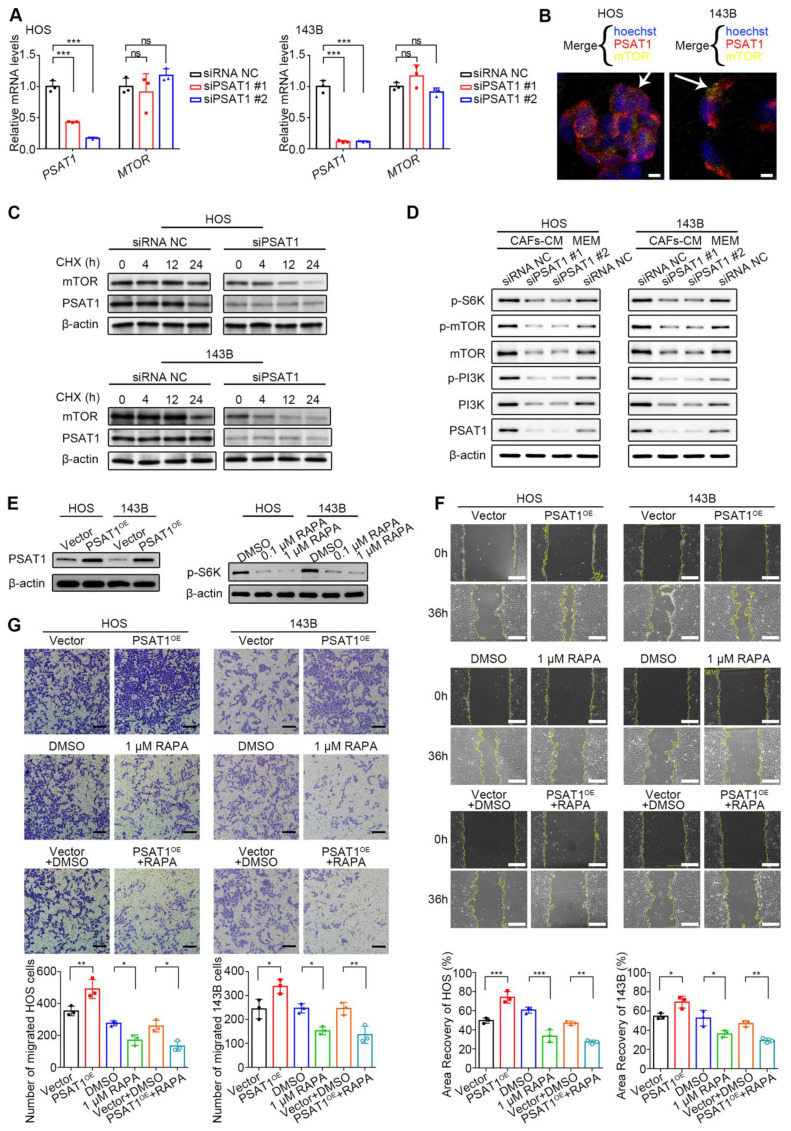
PSAT1 promotes HOS and 143B migration by activating the mTOR/S6K pathway. (A) mTOR transcriptional levels after siPSAT1 interference in HOS and 143B cells by qPCR. (B) The subcellular positions of PSAT1 and mTOR in HOS and 143B cells by immunofluorescence staining. (C) HOS and 143B cells were treated with 100 μg/mL CHX for 0, 4, 12, and 24 hours in the control and siPSAT1 groups, and the mTOR protein levels were analyzed by western blotting. (D) Western blotting analysis of PSAT1, PI3K, p-PI3K, mTOR, p-mTOR, and p-S6K levels after siPSAT1 in HOS and 143B. (E) PSAT1 overexpression (using pcDNA3.1-PSAT1 overexpression plasmid) in HOS and 143B, and mTOR inhibition (using rapamycin) reducing effector protein S6K phosphorylation. (F) Migration of HOS and 143B after PSAT1 overexpression and mTOR pathway inhibition, analyzed by wound-healing assay, scale bars, 200 µm; the indicated results represent the mean ± SD (n=3), * *P* < 0.05, ** *P* < 0.01, *** P < 0.001. (G) Cell migration ability in Transwell assay, scale bars, 100 µm; the indicated results represent the mean ± SD (n=3), * *P* < 0.05, ** *P* < 0.01, *** P < 0.001.

Previous studies have shown that mTOR signaling, which is central to metabolic dysregulation, is directly involved in OS cell migration 26, 27. Based on the GSEA results, it was hypothesized that PSAT1 may promote HOS and 143B cell migration by activating the mTOR pathway. We first evaluated the transcriptional impact of *PSAT1* knockdown on *MTOR* expression via qPCR. No significant alteration in *MTOR* mRNA levels was observed (*P* > 0.05) (Figure 6A). Subsequent immunofluorescence assays demonstrated spatial proximity between PSAT1 and mTOR proteins at the subcellular level in HOS and 143B cells, suggesting potential protein-level regulatory crosstalk (Figure 6B). To assess post-transcriptional regulation, PSAT1-knockdown cells were treated with 100 µg/mL CHX to monitor mTOR protein degradation kinetics. mTOR degradation accelerated progressively with prolonged CHX exposure in PSAT1-knockdown cells, indicating that PSAT1 stabilizes mTOR at the post-transcriptional or post-translation levels (Figure 6C). Thus, the protein levels, as well as phosphorylation levels, of mTOR, its key upstream kinase PI3K, and downstream effector molecule ribosomal protein S6 kinase (S6K) were assessed after PSAT1 knockdown, to investigate whether PSAT1 knockdown inhibits the activity of the PI3K/mTOR/S6K axis. About 6 h after siRNA transfection, the culture media were replaced with CAF-CM or MEM containing 10% FBS for 36 h. Western blotting showed that CAF-CM increased phosphorylation of PI3K and mTOR, whereas PSAT1 knockdown led to marked reductions in both the protein and phosphorylation levels of components of the PI3K/mTOR/S6K axis (Figure 6D). Therefore, PSAT1 can activate the PI3K/mTOR/S6K axis.

PSAT1 was overexpressed using pcDNA3.1-PSAT1 overexpression plasmid (PSAT1^OE^) or mTOR activity was inhibited using rapamycin (RAPA), for the next functional rescue experiments (Figure 6E). RAPA (Selleck, AY-22989) is a potent inhibitor of mTOR/S6K, and was prepared according to the instructions on the Selleck website. At both the 0.1 and 1 µM concentrations, RAPA was found to inhibit S6K phosphorylation (Figure 6E). The wound-healing rate in PSAT1^OE^ HOS and 143B cells was higher than that in vector controls (*P* < 0.05) (Figure 6F), suggesting that elevated PSAT1 leads to OS cell migration. The 1 µM concentration of RAPA resulted in reduced areas of recovery relative to the DMSO controls (*P* < 0.05) (Figure 6F), suggesting that decreased mTOR activity inhibits OS cell migration. To clarify whether PSAT1 promotes OS migration through the mTOR pathway, PSAT1 overexpression combined with mTOR pathway inhibition was performed. It was found that wound healing in the combination group (PSAT1^OE^ + RAPA) was still lower than that of the controls (vector + DMSO) (*P* < 0.05) (Figure 6F). In the Transwell assays, 3*10^4^ OS cells were seeded in the upper compartment. The number of migrated PSAT1^OE^ cells was greater than that seen in the vector controls (*P* < 0.05) (Figure 6G), suggesting that elevated levels of PSAT1 promote migration in OS cells. There were fewer migrated cells in the group treated with 1 µM RAPA relative to the DMSO control (P < 0.05) (Figure 6G), suggesting that reduced mTOR activity inhibits OS cell migration. The migrated cell number was also lower in the PSAT1^OE^ + RAPA group relative to the control vector + DMSO group (*P* < 0.05) (Figure 6G), suggesting that the mTOR pathway functions downstream of PSAT1 in the migration of HOS and 143B cells.

**Figure 7:**
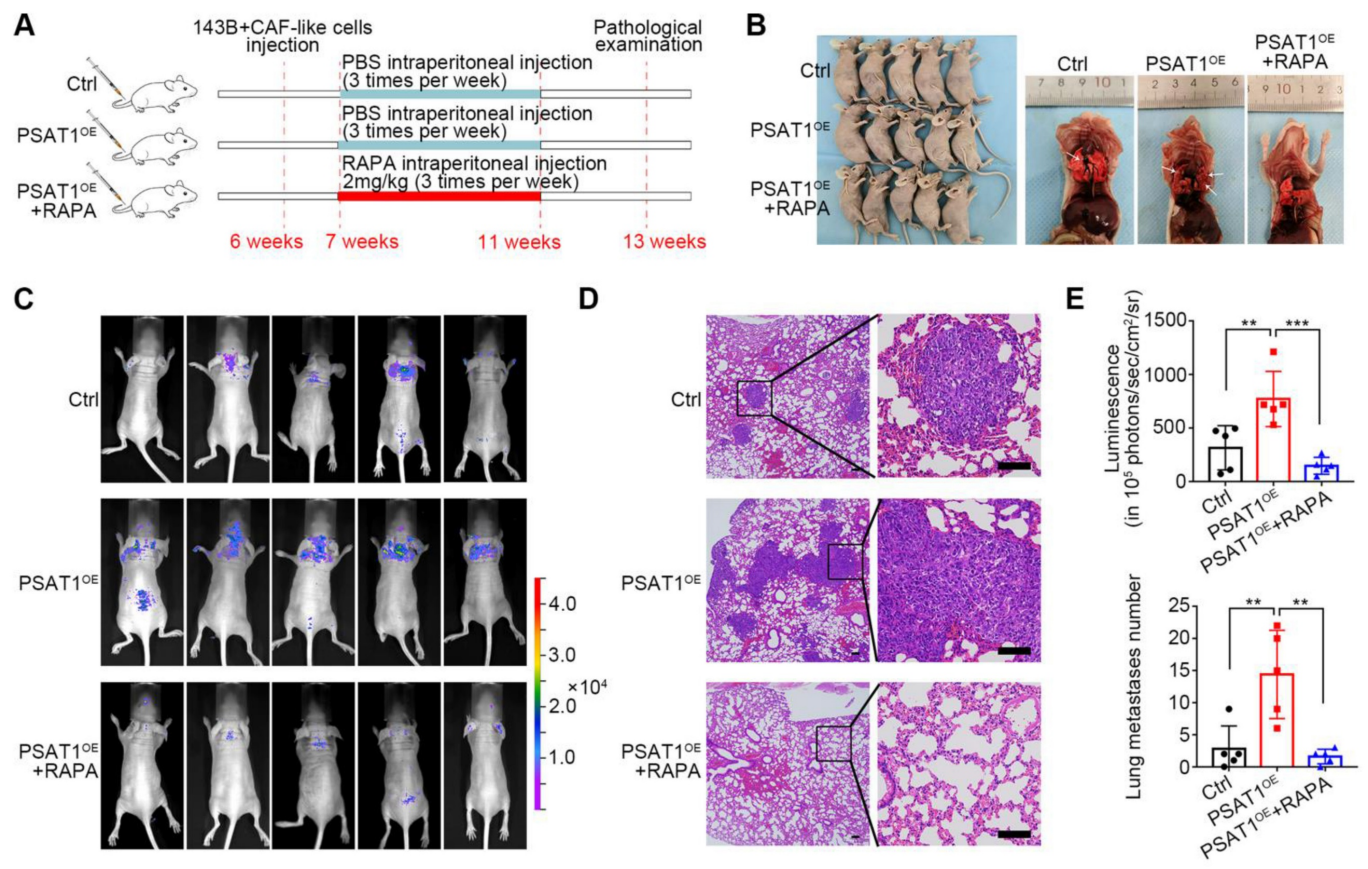
Rapamycin inhibited OS metastasis in 143B lung metastatic model. (A) Administration scheme for 143B lung metastasis model. (B) Photographs of mice in different groups as indicated. (C) Bioluminescence imaging and (D) representative H&E staining of mouse lung metastases, scale bars, 200 µm. (E) Metastasis node numbers in different groups were analyzed, represented by the mean ± SD (n=5), * *P* < 0.05, ** *P* < 0.01, *** *P* < 0.001.

The influence of PSAT1 on metastasis *in vivo* and the potential of RAPA as a treatment for metastatic OS was then assessed in mouse models using PSAT1^OE^ or vector 143B cells (Figure 7A). Following intraperitoneal injection of PBS or 2 mg/kg RAPA, the lungs of the mice were collected and analyzed (Figure 7B). Bioluminescence imaging and H&E staining of mouse lung metastases further showed that the mice given PSAT1^OE^ 143B cells had greater numbers of lesions, while RAPA treatment reduced metastasis (Figure 7C, D). Consistent with this result, elevated PSAT1 levels promoted OS metastasis to the lungs, which was reduced by RAPA (Figure 7E). These data suggested that PSAT1 contributed to OS metastasis which could be alleviated by mTOR inhibition *in vivo*.

## Discussion

CAFs have been demonstrated to promote cancer metastasis and are also recognized as potential targets for anti-tumor therapy 9-11. However, approaches aimed at depleting CAFs using biomarkers have been linked to various adverse outcomes and worse survival, impeding their clinical use 28, 29. Blocking the crosstalk between CAFs and tumor cells or inducing reprogramming of the tumor by CAFs would be expected to be more effective. Here, we report that *in vitro*-activated CAF-like cells promoted OS metastasis by reprogramming the metabolic profile of the tumor cells and increasing the levels of the serine metabolism-associated enzyme PSAT1. Consistent with these findings, recent studies have indicated that CAFs are influenced by tumor cells and the crosstalk between them initiates the metabolic reprogramming of tumor cells to adapt to the hostile microenvironment 30, 31. Kerk *et al.* reported that CAFs can activate glutamate-oxaloacetate transaminase 2 (GOT2) in pancreatic ductal adenocarcinoma (PDAC) by supplying pyruvate, thereby rescuing GOT2 deletion-mediated growth inhibition in PDAC cells 30. Here, we also focused on the downstream pathways associated with the overactivated metabolic enzymes; consequently, we propose a feasible strategy for targeting OS metastasis that avoids the problem of severe adverse effects caused by the direct removal of CAFs, by blocking the downstream effects of tumor metabolic reprogramming induced by CAFs. The present study examined the metabolic pathways altered in HOS cells after co-culture with CAF-like cells using LC-MS and confirmed the key function of PSAT1 in OS metastasis through activation of the mTOR/S6K axis.

CAFs are considered stromal cells that are activated by tumor cells, and their precursor cells are mostly NFs and MSCs. Currently, there are two major methods for isolating CAFs, including, primary culture of cells collected from tumor tissues and the activation of precursor cells into CAF-like cells *in vitro* 32-35. Mishra *et al.* performed whole-genome sequencing of precursor MSCs, *in vitro*-induced CAF-like cells, and intratumor primary CAFs, observing similar gene expression profiles in both vitro-activated CAF-like cells and primary CAFs obtained *in vivo*; these differed markedly from the profiles of precursor MSCs, thus confirming the similarity between CAF-like cells and intratumor CAFs 34. It has also been reported that NFs can be converted to CAF-like cells by OS cells *in vitro* 11, 21, 22. In this study, we obtained CAF-like cells by co-culture of HOS/143B-CM and WI-38 cells (Supplementary Figure 1A), finding that the co-cultured WI-38 cells expressed the documented CAF biomarkers as well as increased levels of the inflammatory factors IL-1β, IL-6, and TGF-β, thus verifying that the cells were CAF-like cells (Figure 1A-D). The identity of the CAF-like cells was also verified by evaluation of the phosphorylation of components of the IL-6/STAT3 and TGF-β/Smad2 pathways (Figure 1H). Consistent with previous evidence, CAFs show a markedly greater secretion of inflammatory factors as well as hyperactivation of inflammatory pathways. Mazumdar *et al.* verified that the conversion process of NFs to CAFs is TGF-β-dependent in OS, as phosphorylation of Smad2, a downstream effector of TGF-β, was increased while the application of the TGF-β1 receptor inhibitor, SB-431542, or the use of CRISPR-Cas9-mediated deletion of the *TGFB1* gene significantly interfered with the conversion of NFs to CAFs 21. Another study induced the conversion of MSCs to CAFs using the OS cell line U2OS, with transcriptomic sequencing results showing that this process was associated with overexpression of IL-6 and phosphorylation of STAT3, whereas blocking of the IL-6/STAT3 axis in MSCs rescued the phenotypic transformation of CAFs 22.

Abundant evidence has demonstrated the promotion of CAFs for tumor migration 11, 36. In this study, CAF-CM was used to treat HOS and 143B cells, and CCK-8, clonogenic survival, wound-healing, and Transwell assays were performed, showing that the CAF-like cells had no significant effects on OS proliferative activity (Supplementary Figure 3), although they markedly promoted both cell migration (Figure 2 A, B) and the EMT (Figure 2 C). In addition, *in vivo* experiments also showed greater numbers of metastatic lesions in the lungs of nude mice treated with HOS in the CAFs cell-treated group relative to the controls (Figure 2 D, E). Recent studies have increasingly recognized that all stages of tumor metastasis rely on metabolic reprogramming 37. During the pre-invasion phase, greater amounts of CO_2_, lactate, and organic acids are released from metabolically active tumor cells, leading to acidification of the extracellular environment and degradation of the ECM, thus facilitating tumor invasion 38. When tumor cell migration occurs, upregulation of metabolic enzymes such as uridine 5'-diphosphate-glucose 6-dehydrogenase and asparagine synthetase enhances the expression of EMT-associated proteins 39, 40. Within the circulatory system, tumor cells adjust their metabolism to alleviate oxidative stress, such as through the synthesis of glutathione to maintain the redox balance 41. During organ colonization and the development of macroscopic metastases, reactivation of many metabolic pathways occurs to facilitate the growth of the metastatic foci 42. Previous studies have also concluded that CAFs are major regulators that shape tumor metabolism and mediate the dysregulation of tumor glucose, amino acid, and lipid metabolic pathways, thereby promoting malignancy 14. For example, CAFs are involved in the tumor “reverse Warburg effect” involving aerobic glycolysis, which provides tumor cells with supplies of energy, lactate, and pyruvate 43. In addition, recent studies have shown that CAFs also alter amino acid metabolism in tumor cells by up-regulating transcription factors, producing soluble modulatory factors 20; importantly, dysregulated amino acid metabolism also plays a major part in cancer by participating in the maintenance of redox homeostasis, energy generation, synthesis of biomolecules, epigenetic modifications, drug resistance, and immune escape 24, 44. To our knowledge, the profiles of CAFs in relation to OS metabolism lack a comprehensive evaluation, and existing studies have only explored several metabolic enzymes of CAFs affecting OS recurrence and metastasis 11, 20. The present study bridges the research gap in this regard. The results of the metabolomics analysis, in both the positive ion mode (Supplementary Table 2) and negative ion mode (Supplementary Table 3), showed that amino acid metabolic pathways accounted for the majority of the enriched pathways, while the KEGG and MSEA enrichment results indicated that serine and methionine metabolism in OS cells were significantly impacted by CAF-like cells (Figure 3D, E). The serine synthesis pathway (SSP) is closely associated with methionine metabolism, and both are involved in the one-carbon cycle of aberrant tumor metabolism 45, 46. The SSP includes three key enzymes, namely, PHGDH, PSAT1, and phosphoserine phosphatase (PSPH). The glycolysis intermediate metabolite, 3-phosphoglycerate, is converted to 3-phosphohydroxypyruvate by PHGDH and undergoes further breakdown to 3-phosphoserine by PSAT1, followed by dephosphorylation by PSPH, resulting in the production of serine to participate in important biological processes such as protein biosynthesis and lipid metabolism 47. In addition, exogenous serine can undergo conversion to glycine mediated by serine hydroxymethyl transferase (SHMT), resulting in a carbon unit that can take part in the one-carbon cycle involved in nucleotide biosynthesis 45. In turn, combination of the one-carbon metabolic pathway with the folate and methionine metabolism metabolic cycles can contribute to nucleotide synthesis and the maintenance of redox balance within the cell. One-carbon metabolism is required for producing S-adenosyl methionine (SAM), a metabolite necessary for histone and DNA methylation, enhancing methylation and altering the epigenetic landscape in tumor cells 45-47. In our study, qPCR was utilized to verify the expression of the above key enzymes, which showed that PSAT1 levels differed significantly between the groups (Figure 3F), suggesting that CAF-like cells promoted increased expression of PSAT1 in OS cells.

High PSAT1 expression has been linked with poor prognosis in many tumors 48-50. A pan-cancer analysis of PSAT1 suggested associations between PSAT1 levels and immune infiltration, specifically, of CD8+ T cells, CD4+ T cells, macrophages, neutrophils, and dendritic cells 51. In the present study, we performed bioinformatics analysis using public databases (Supplementary Figure 6), and also evaluated the primary and metastatic foci of tissue samples from patients with OS (Figure 4, Supplementary Figure 5), which demonstrated that the levels of PSAT1 were elevated in OS tissues and positively correlated with poor survival. Increased PSAT1 levels were also positively linked to CAF abundance (Figure 3F, Figure 4E). Then, si-PSAT1, wound-healing, and Transwell were performed on cells co-cultured with CAF-CM, indicating markedly reduced OS cell migration (Figure 5B, C) and EMT suppression (Figure 5D) in the PSAT1-knockdown group. Therefore, CAF-like cells can promote HOS and 143B cell migration and the EMT via PSAT1. PSAT1 represents a major rate-limiting enzyme in the SSP, and is responsible for maintaining normal serine biosynthesis of serine by catalyzing the reversible conversion of 3-phosphohydroxypyruvate to 3-phosphoserine, as well as also facilitating the production of α-ketoglutarate to participate in the tricarboxylic acid (TCA) cycle 50, 52. However, our study found that excess serine did not promote HOS cell migration (Supplementary Figure 9), suggesting that PSAT1 may mediate OS metastasis through other mechanisms. Luo *et al.* observed that PSAT1 functioned as a non-metabolically regulated protein in EGFR inhibitor-resistant cells, synergistically regulating the ROS/JNK/c-Jun and IQGAP1-STAT3 axes to promote tumor metastasis 25. In other studies, PSAT1 was shown to interact with pyruvate kinase M2 (PKM2) and promote nuclear translocation of PKM2 to contribute to the migration of lung cancer cells 53, 54. PSAT1 also interacts with the codon 72 polymorphism of p53 (p53^72p^) competing with PPARγ coactivator-1α for binding to p53^72p^ and thereby dissociating the former and activating oxidative phosphorylation and the TCA, whereas PSAT1 knockdown significantly reduced migration in hepatocellular carcinoma cells 55. Meanwhile, PSAT1 has also been found to act as a target of several long non-coding RNAs, which has been suggested to promote metastasis 56, 57. Therefore, PSAT1 exhibits potential convergence of metabolic and non-metabolic functions. Future studies warrant further investigation into the plasticity of PSAT1 as a signaling scaffold for effector proteins (such as PKM2 and mutant p53), and its regulatory role in redox-sensitive crosstalk within kinase signaling networks. The present study focused more on the non-metabolic regulatory functions of PSAT1 and performed GSEA pathway enrichment in pan-cancer. The results suggested that mTOR signaling, among other pathways, was widely enriched (Supplementary Figure 10). We also observed the enrichment of the E2F transcription factor (NES=2.308), MYC target (NES=2.017), and mTOR signaling (NES=1.906) in SARC sarcoma (all *P* < 0.05).

mTOR was identified as a target of RAPA in 1994, and its pivotal role in regulating cellular metabolism, autophagy, and growth is well-documented 58. In response to upstream signals, mTOR can form two complexes that differ in both structure and function, namely, mTOR complex 1 (mTORC1) (containing mTOR, Raptor, MLST8, PRAS40, and Deptor molecules) and mTORC2 (containing mTOR, Rictor, mSIN1, Protor-1, mLST8, and Deptor molecules) 59. The KEGG database recognizes six major regulatory pathways upstream of mTORC1, namely, those involved in amino acid metabolism, energy deficiency, hypoxia, and the Wnt, TNF-α, and insulin pathways; whereas mTORC2 is mainly involved in pathways associated with insulin signaling. Activation of mTORC1 and mTORC2 is closely related to PI3K phosphorylation and activation 58, 59. mTOR-associated activities are aberrantly activated in approximately 30% of tumors 60, including OS 61, 62. Consistent with our results, previous studies have suggested that the mTOR pathway is involved in proliferation, migration, apoptosis, autophagy, and malignancy-associated behaviors in OS, with dysregulation of upstream PI3K due to increased levels of HER-2 and insulin-like growth factor receptor, resulting in over-activation of the downstream components S6K and 4E-BP, leading to poor OS prognosis 61-63. In this study, mTOR signaling was found to function downstream of PSAT1 in CAF-like cells, influencing OS metastasis, while the mTOR inhibitor RAPA exerted an inhibitory effect on OS metastasis (Figure 6F and G, Figure 7). It is suggested that targeting the PSAT1 and mTOR pathways is a promising approach for treating metastatic OS. However, the regulatory crosstalk between the mTOR pathway and PSAT1 requires further investigation. There is also a lack of PSAT1-targeted drugs and related clinical trials, with only one registered clinical trial (NCT02103920) for retrospective expression detection of glycine/serine metabolism pathway molecules (e.g., PSPH, PSAT1, SHMT1, and GLDC) in solid tumors such as colorectal, lung, and breast cancers. It is thus feasible to develop drugs targeting PSAT1. Targeting PSAT1 with small-molecule inhibitors, either alone or in combination with mTOR pathway blockers, may improve therapeutic outcomes of metastatic OS, which will be prioritized in our preclinical studies.

While this study provides novel insights into CAF-driven OS metastasis, several limitations should be acknowledged. First, although our clinical cohort and public database analyses support the prognostic value of PSAT1 in OS, future multi-center investigations incorporating larger sample sizes and subtype stratification will be essential to validate these findings and establish clinical applicability. Second, while CAF-CM induced pro-metastatic metabolic reprogramming in OS cells, the specific mediators responsible for these effects were not fully dissected; future proteomic profiling of CAF-CM will help identify dominant drivers. Third, although we demonstrated that PSAT1 stabilizes mTOR protein to activate S6K, the precise molecular events linking PSAT1 to mTOR require further validation using techniques like mass spectrometry assays. Lastly, our *in vivo* model employed tail vein injection to simulate lung metastasis, whereas spontaneous lung metastasis model of OS would better recapitulate the clinical progression. Addressing these limitations in future studies will deepen the understanding of the CAF-PSAT1-mTOR axis and its therapeutic potential.

## Supplementary Material

Supplementary figures and tables.
