# Supplementary material for: Cancer-associated Fibroblast-like Cells Promote Osteosarcoma Metastasis by Upregulation of Phosphoserine Aminotransferase 1 and Activation of the mTOR/S6K Pathway
Source: Int J Biol Sci. 2025 Jun 20;21(9):4153–71. doi: 10.7150/ijbs.109169 (PMC12223780; doi:10.7150/ijbs.109169)
Supplement: Supplementary file 1 — Supplementary figures and tables. [file ijbsv21p4153s1.pdf]

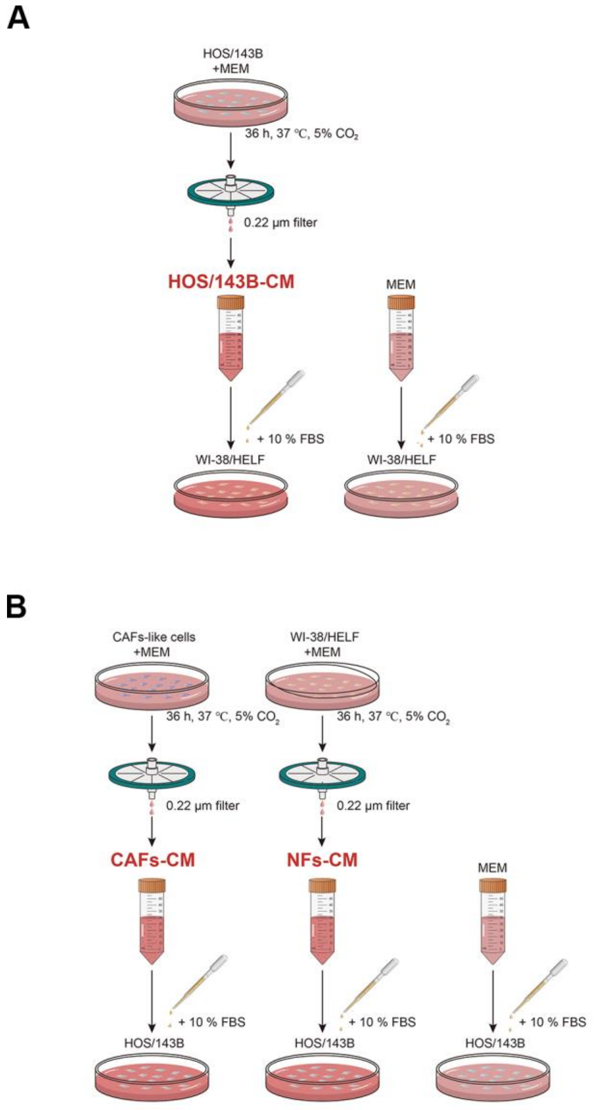

Supplementary Figure 1. Schematic diagram of the CM preparation process. (A) Tumor cells CM preparation and the method of fibroblast activation. (B) Fibroblasts CM preparation and the method of co-culture with tumor cells in vitro.

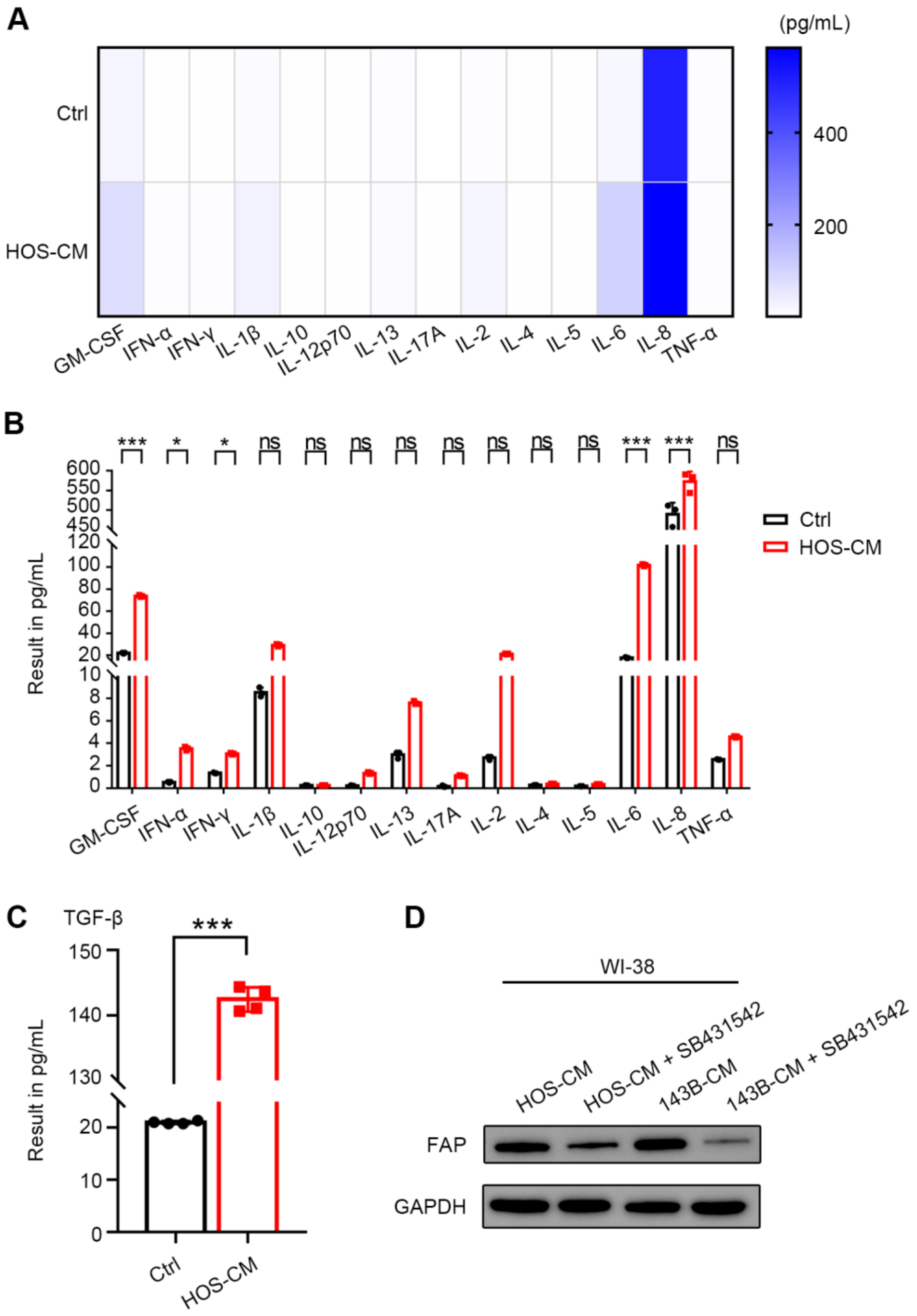

Supplementary Figure 2. The secretory profile of HOS cells mediated the conversion of WI-38 cells into CAF-like cells. (A) Heatmap depicting cytokine secretion in Ctrl (WI-38-CM) and HOS-CM assessed by a 14-plex multiplex cytometric bead array panel. (B) The t-test was used to evaluate the differences between the two groups, the values are expressed as the mean ± SD (n=3), ns: not significant, * $P < 0.05$, ** $P < 0.01$, *** $P < 0.001$. (C) The secretion levels of TGF-β in Ctrl (WI-38-CM) and HOS-CM by ELISA, the values are expressed as the mean ± SD (n=4), ns: not significant, * $P < 0.05$, ** $P < 0.01$, *** $P < 0.001$. (D) The expression level changes of activated marker FAP in CAF-like cells after TGF-β inhibitor treatment (10 μM SB431542) by western blotting.

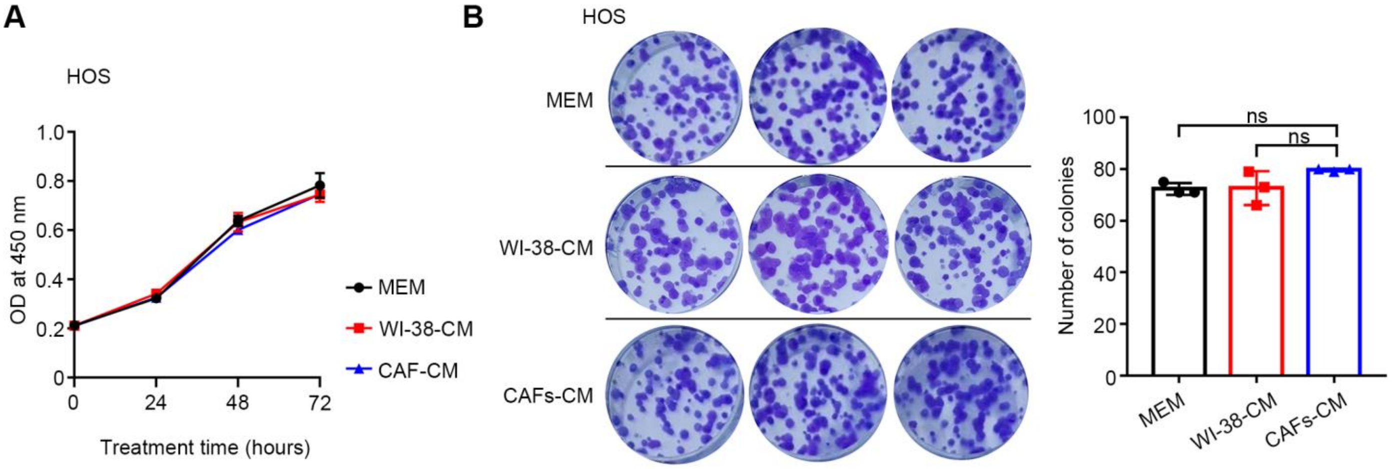

Supplementary Figure 3. Effects of CAF-like cells on the proliferative activity of HOS. (A) Number of viable cells in the CCK-8 assay was displayed as OD value at 450 nm. (B) Cell proliferation capacity was analyzed by clonogenic survival assay, and expressed as the number of colonies formed on day 10. HOS was treated with CAFs-CM, while the control group was the WI-38-CM and the MEM group. The values are expressed as the mean ± SD (n=3), ns: not significant, * $P < 0.05$, ** $P < 0.01$, *** $P < 0.001$.

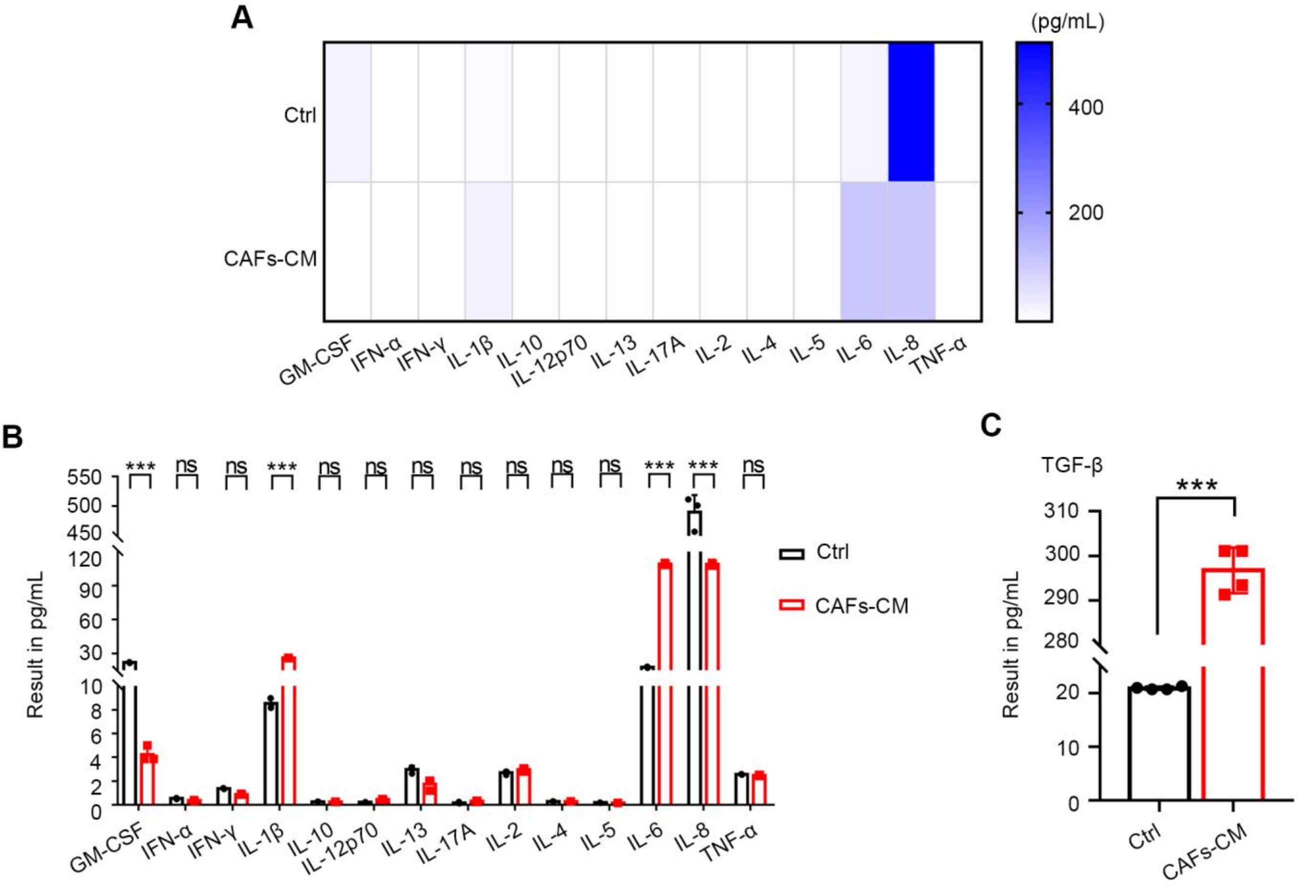

Supplementary Figure 4. The secretory profile of CAF-like cells. (A) Heatmap depicting cytokine secretion in Ctrl WI-38-CM and CAFs-CM assessed by a 14-plex multiplex cytometric bead array panel. (B) The t-test was used to evaluate the differences between the two groups, the values are expressed as the mean ± SD (n=3), ns: not significant, * $P < 0.05$, ** $P < 0.01$, *** $P < 0.001$. (C) The secretion levels of TGF-β in Ctrl WI-38-CM and CAFs-CM by ELISA, the values are expressed as the mean ± SD (n=4), ns: not significant, * $P < 0.05$, ** $P < 0.01$, *** $P < 0.001$.

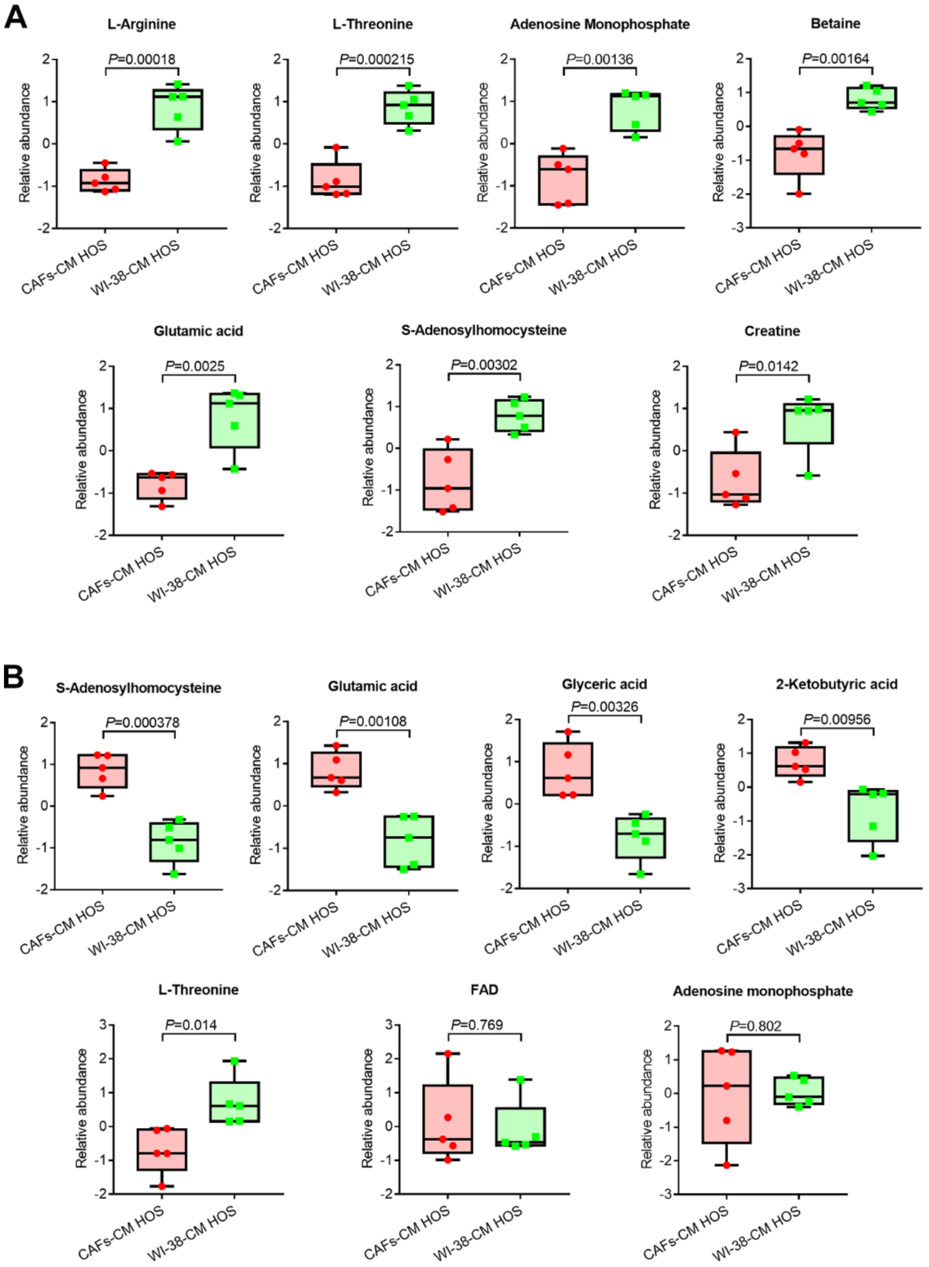

Supplementary Figure 5. Relative abundance of detected metabolites involved in Glycine and Serine Metabolism pathway using (A) positive ion mode and (B) negative ion mode.

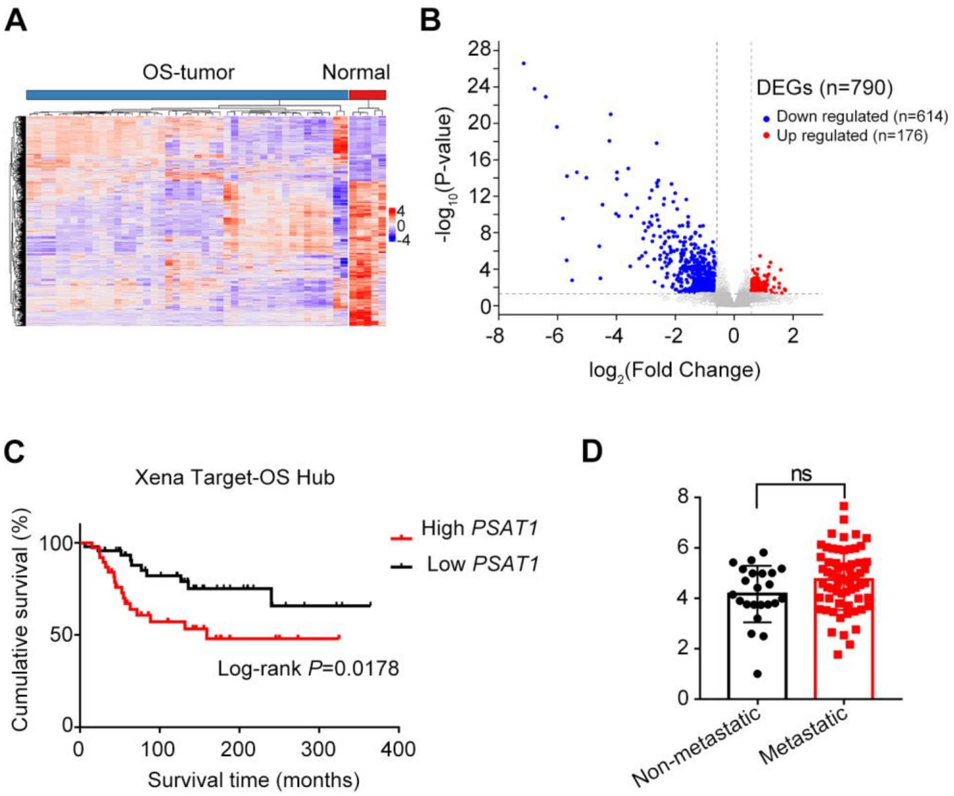

Supplementary Figure 6. Bioinformatics analysis of PSAT1 expression in OS. (A) Heatmap of transcriptome gene in GEO database between OS tumors and normal tissues. The color bars represent the $\log_{10}$ value of the significance of each gene. (B) Volcano plot of differentially expressed genes (DEGs) in GEO database between OS tumors and normal tissues. (C) Kaplan-Meier curves for OS patients in the Xena Target-Dataset between high *PSAT1* and low *PSAT1* groups. (D) *PSAT1* gene expression in the Xena Target-Dataset between Non-metastatic (n=23) and metastatic (n=62) OS patients.

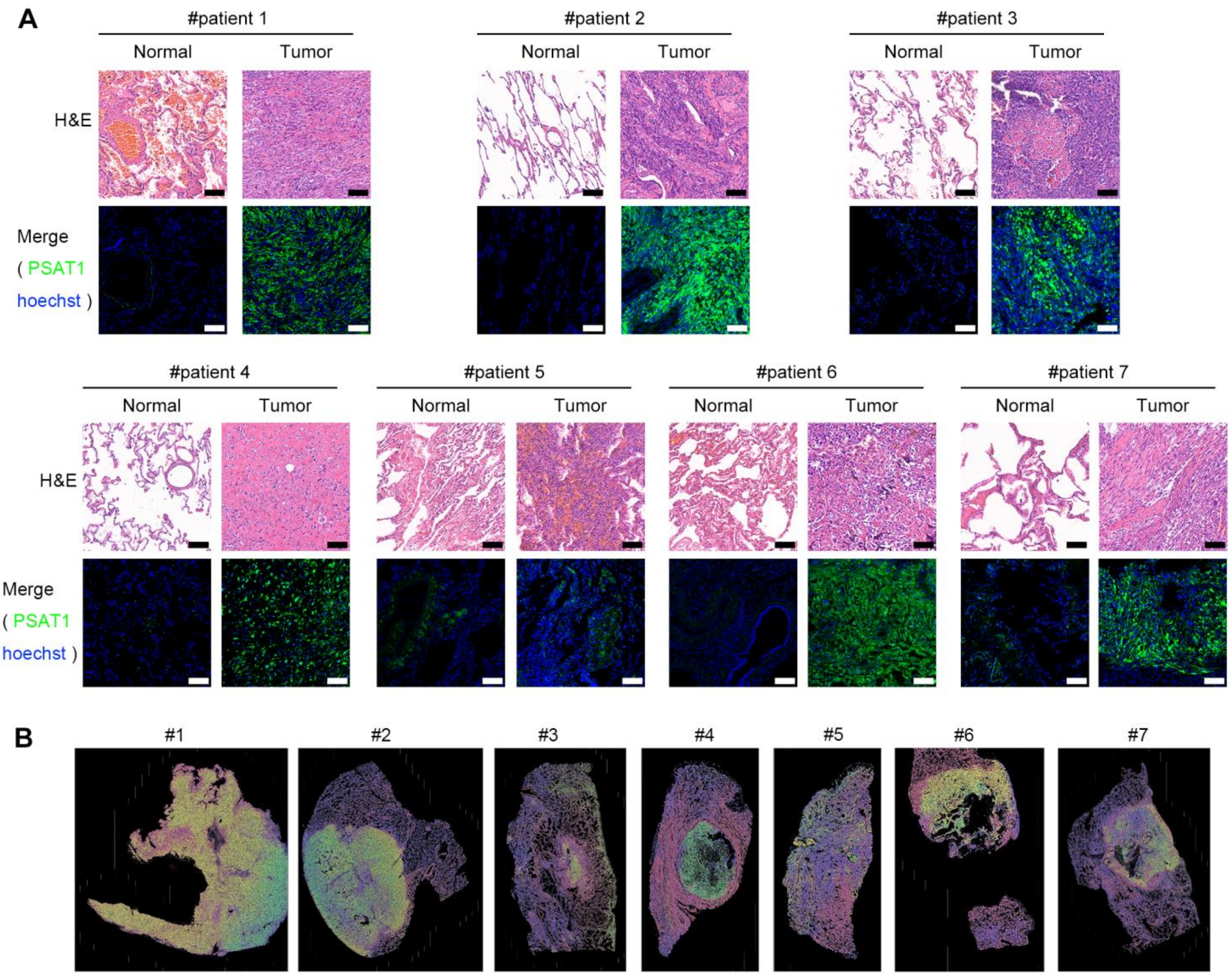

Supplementary Figure 7. (A) H&E staining and PSAT1 immunofluorescence staining in 7 cases of OS lung metastases and paired normal lungs, scale bars, 50 μm. (B) Panoramic scans of immunofluorescence staining demonstrating the expression of PSAT1 and α-SMA in 7 cases of OS lung metastases. (green, PSAT1; red, α-SMA; blue, hoechst; 6×magnification).

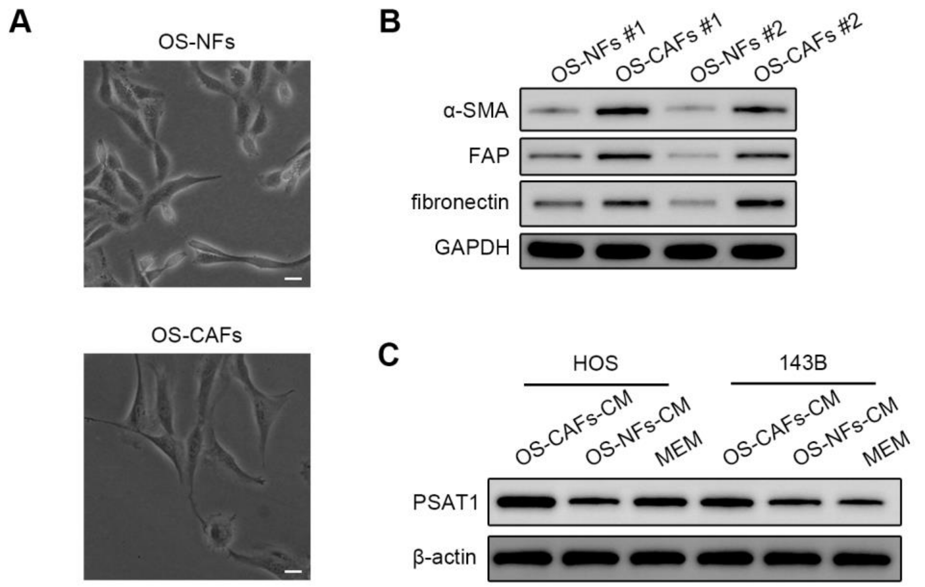

Supplementary Figure 8. Isolation of primary CAFs and NFs in OS patients and their influence on PSAT1 levels in HOS and 143B. (A) The morphological images of primary NFs and CAFs in OS patients, scale bars, 50 µm. (B) The protein level of CAF biomarkers in primary NFs and CAFs by western blotting. (C) The protein level of PSAT1 in HOS and 143B cells after co-culturing with primary CAFs-CM or NFs-CM.

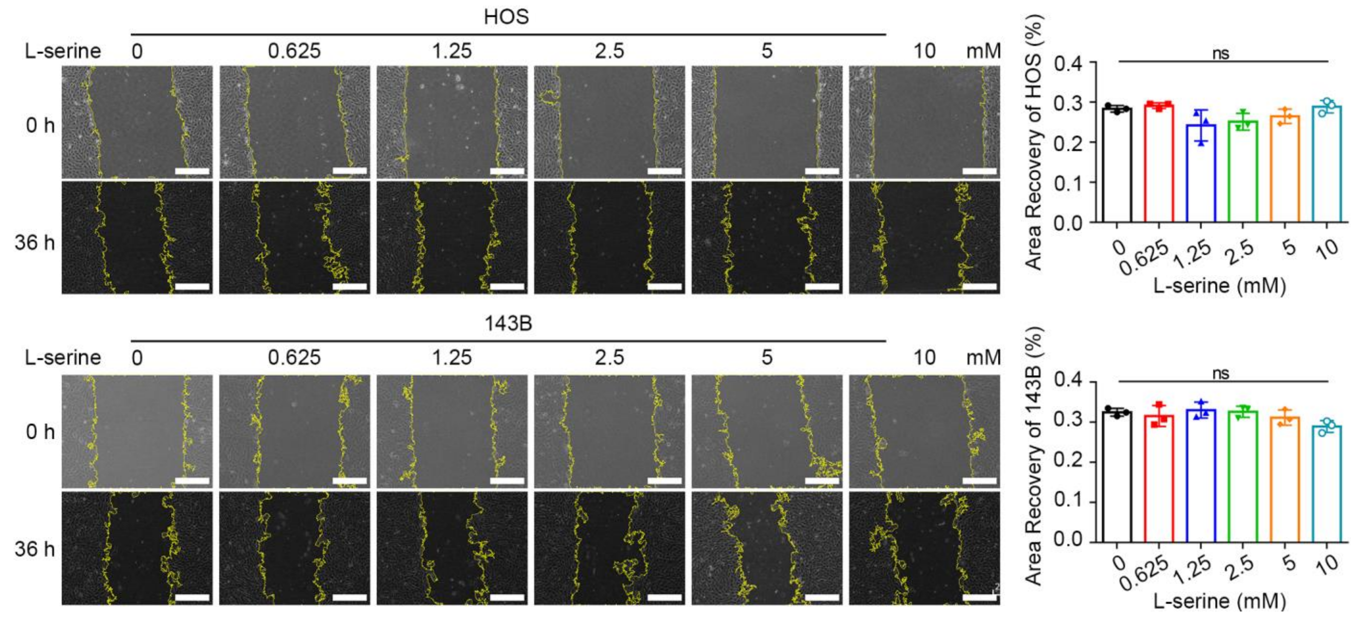

Supplementary Figure 9. Migration of HOS and 143B at different serine concentrations was analyzed by wound-healing assay and expressed as area recovery rate, scale bars, 200 μm; the indicated results represent the mean ± SD (n=3), ns: not significant.

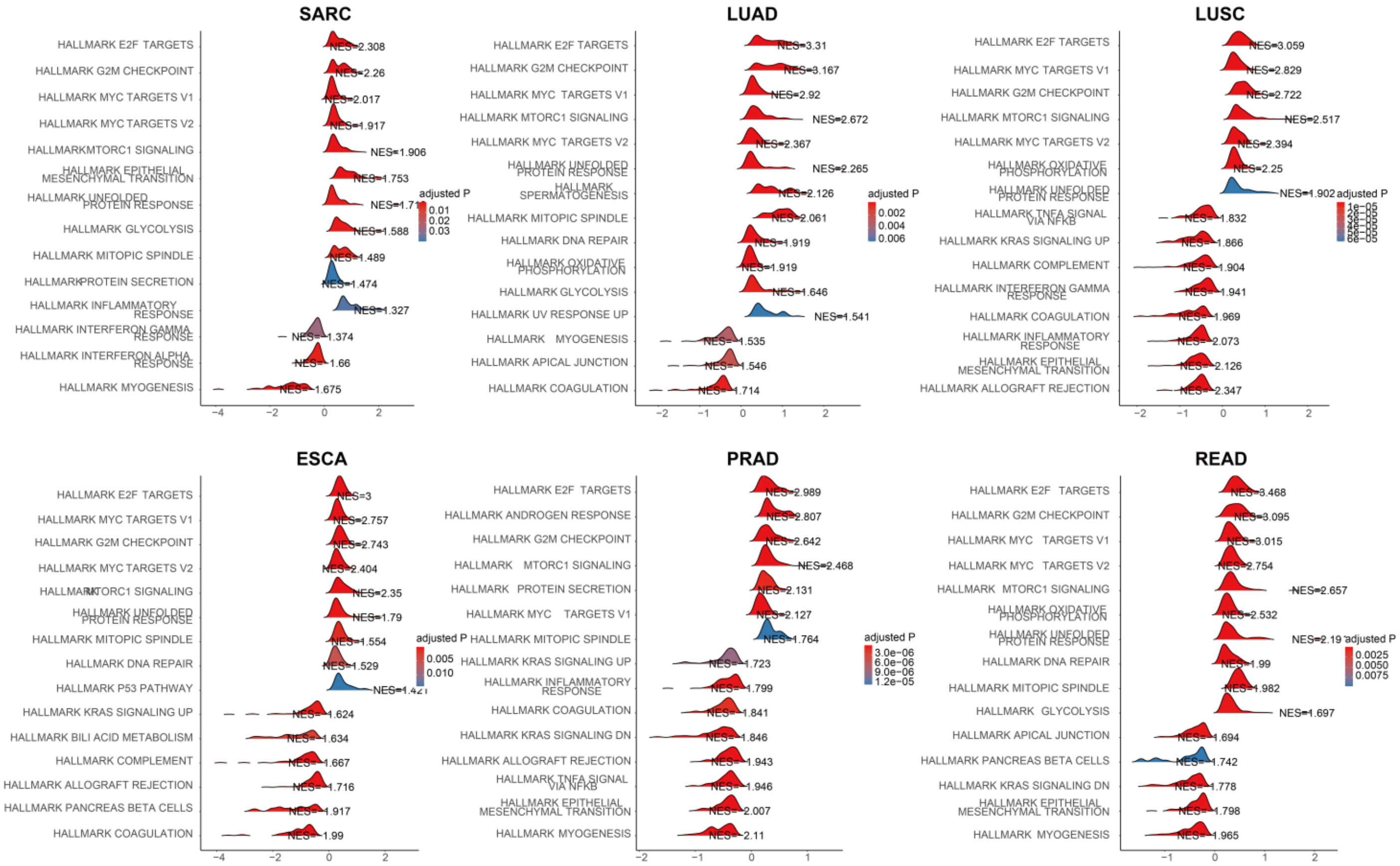

Supplementary Figure 10. GSEA analysis of PSAT1 for signaling pathway enrichment in pan-cancer. SARC, sarcoma; LUAD, lung adenocarcinoma; LUSC, lung squamous cell carcinoma; ESCA, esophageal carcinoma; PRAD, prostate adenocarcinoma; READ, rectum adenocarcinoma.

Table S-1. Sequences of primer used in qPCR analysis.

Gene name	Forward primer	Reverse primer
GAPDH	TCGGAGTGAACGGATTTGGC	CCAGCATCACCCCACTTGAT
ACTB	GGCTGTGCTATCCCTGTACG	AGGGCATACCCCTCGTAGAT
ACTA2	GAAGAAGAGGACAGCACTGCCTT	TATCGGGTACTTCAGGGTCAGGA
FN1	TAGAATTGGAGACACCTGGAGCA	TGTCTGTGACACAGTGGCCATAG
FAP	CATCTATGACCTTAGCAATGGAG	TAGGAGACCACCAGAGAGCATAT
IL6	CCTGGTGAAAATCATCACTGGTCT	ATGAGATGAGTTGTCATGTCCTGC
IL1B	GACTTGTTCTTTGAAGCTGATGGC	GTCATTCTCCTGGAAGGTCTGTG
TGFB1	GCTCCTGTGACAGCAGGGATAAC	TGGAGCTGAAGCAATAGTTGGTG
PHGDH	AATCTGCGGAAAGTGCTC	ATTTGCCGTCCTTCATCG
PSAT1	CAGTTCAGTGCTGTCCCC	TGAGGTTCCAGGTGCTTG
PSPH	GACAGCACGGTCATCAGA	GCTCCTGTAGGCGACTTA
SHMT1	CTTCTACAGGAAAGGAGTGA	CCTCTGTGAATAAAGTGGG
SHMT2	CAGTTCCGTGAGGATGAC	CAGACGCTGACTTGTTTC
GLDC	CACTATCCGAGCCTACTTA	GGTTCTCCTTGTGCTTATC
MAT2A	CCAAACTGGCAGAACTAC	GACCTGAACAAGAACCCT
AHCY	GACAAACTGCCCTACAAA	CCCGTCGTCCAGAATCAT
MTOR	CCTCCATCCACCTCATCA	GACGCCAAGACACAGTAG

Table S-2. KEGG analysis of HOS metabolites affected by CAFs-like cells in positive ion mode.

KEGG pathway	P value	FDR	Pathway impact
Glycolysis or Gluconeogenesis	7.27E-05	0.002837	0
Thiamine metabolism	0.000941	0.016207	0.12481
Tryptophan metabolism	0.001247	0.016207	0.2205
Riboflavin metabolism	0.002155	0.01805	0.14504
Phenylalanine, tyrosine and tryptophan biosynthesis	0.00268	0.01805	0.008
Nitrogen metabolism	0.002957	0.01805	0
Tyrosine metabolism	0.003906	0.01805	0.04724
Ubiquinone and other terpenoid-quinone biosynthesis	0.003906	0.01805	0
Phenylalanine metabolism	0.004165	0.01805	0.15431
Aminoacyl-tRNA biosynthesis	0.005064	0.01975	0.11268
Glycine, serine and threonine metabolism	0.005894	0.020151	0.12069
Cysteine and methionine metabolism	0.0062	0.020151	0.10058
Nicotinate and nicotinamide metabolism	0.012728	0.03398	0.05492
Amino sugar and nucleotide sugar metabolism	0.013887	0.03398	0.00265
Primary bile acid biosynthesis	0.013941	0.03398	0.05524
Steroid hormone biosynthesis	0.013941	0.03398	0.00391
Arginine and proline metabolism	0.018744	0.043002	0.33494
D-Arginine and D-ornithine metabolism	0.022475	0.048696	0
Glutathione metabolism	0.029565	0.060686	0.08349
Pantothenate and CoA biosynthesis	0.033284	0.06313	0.18014
Valine, leucine and isoleucine degradation	0.037127	0.06313	0
Propanoate metabolism	0.037127	0.06313	0
Fatty acid metabolism	0.037231	0.06313	0
Valine, leucine and isoleucine biosynthesis	0.045769	0.074374	0.01325
beta-Alanine metabolism	0.060844	0.093312	0.06625
Pyrimidine metabolism	0.062208	0.093312	0.20695
Purine metabolism	0.068627	0.099128	0.1455
Sphingolipid metabolism	0.075859	0.10566	0
Histidine metabolism	0.083745	0.10774	0.14039
Alanine, aspartate and glutamate metabolism	0.088401	0.10774	0.17664
D-Glutamine and D-glutamate metabolism	0.088401	0.10774	0.1123
Butanoate metabolism	0.088401	0.10774	0
Porphyrin and chlorophyll metabolism	0.10438	0.12336	0
Caffeine metabolism	0.13357	0.15321	0.22419
Glycerophospholipid metabolism	0.17836	0.19875	0.07598
Taurine and hypotaurine metabolism	0.70329	0.7619	0.08094
Lysine degradation	0.86266	0.86266	0.14675
Lysine biosynthesis	0.86266	0.86266	0.09993
Biotin metabolism	0.86266	0.86266	0

Table S-3. KEGG analysis of HOS metabolites affected by CAFs-like cells in negative ion mode.

KEGG pathway	P value	FDR	Pathway impact
Alanine, aspartate and glutamate metabolism	3.21E-05	0.000595	0.65148
Arginine and proline metabolism	3.36E-05	0.000595	0.04505
Tryptophan metabolism	3.43E-05	0.000595	0.10853
Nitrogen metabolism	4.61E-05	0.0006	0.00067
D-Glutamine and D-glutamate metabolism	6.73E-05	0.000626	0.13904
Aminoacyl-tRNA biosynthesis	7.36E-05	0.000626	0.11268
Histidine metabolism	8.43E-05	0.000626	0.14039
Glutathione metabolism	0.000147	0.000953	0.25028
Porphyrin and chlorophyll metabolism	0.000298	0.001724	0
Cysteine and methionine metabolism	0.000567	0.002948	0.10793
Primary bile acid biosynthesis	0.00066	0.00312	0.01695
Taurine and hypotaurine metabolism	0.001113	0.004823	0.33094
Phenylalanine, tyrosine and tryptophan biosynthesis	0.002599	0.010395	0.00738
Nicotinate and nicotinamide metabolism	0.004682	0.017389	0
Lysine biosynthesis	0.00561	0.019449	0.06769
Cyanoamino acid metabolism	0.00665	0.021611	0
Glycine, serine and threonine metabolism	0.010021	0.030654	0.09708
Caffeine metabolism	0.021083	0.060907	0
Butanoate metabolism	0.023605	0.064602	0.0611
Glycerolipid metabolism	0.028366	0.073752	0.0206
Pyrimidine metabolism	0.035066	0.086831	0.23017
Purine metabolism	0.040553	0.095852	0.2306
Propanoate metabolism	0.048762	0.11024	0.03027
beta-Alanine metabolism	0.054175	0.11738	0
Phenylalanine metabolism	0.074671	0.14766	0
Citrate cycle (TCA cycle)	0.078351	0.14766	0.14968
Tyrosine metabolism	0.078994	0.14766	0.04724
Pantothenate and CoA biosynthesis	0.079511	0.14766	0.18014
Glyoxylate and dicarboxylate metabolism	0.098541	0.17669	0.04796
Pentose and glucuronate interconversions	0.12094	0.20963	0.00638
Pentose phosphate pathway	0.17251	0.28937	0.20621
Amino sugar and nucleotide sugar metabolism	0.22891	0.37198	0.21451
Galactose metabolism	0.24816	0.39105	0.07255
Valine, leucine and isoleucine degradation	0.2811	0.41836	0.0421
Fatty acid metabolism	0.28963	0.41836	0.02959
Fatty acid elongation in mitochondria	0.28963	0.41836	0
Steroid hormone biosynthesis	0.30591	0.4288	0
Starch and sucrose metabolism	0.31336	0.4288	0.29376
Arachidonic acid metabolism	0.33706	0.44942	0.21669
Lysine degradation	0.35158	0.45706	0.01563
Fructose and mannose metabolism	0.3876	0.48117	0.02948
Glycolysis or Gluconeogenesis	0.39529	0.48117	0
alpha-Linolenic acid metabolism	0.40479	0.48117	0.20335
Valine, leucine and isoleucine biosynthesis	0.40714	0.48117	0.08003
Thiamine metabolism	0.459	0.5304	0
Riboflavin metabolism	0.47154	0.53305	0
Fatty acid biosynthesis	0.68117	0.75364	0

Ascorbate and aldarate metabolism	0.73537	0.79666	0.00802
Ubiquinone and other terpenoid-quinone biosynthesis	0.77339	0.82074	0.0368
Glycerophospholipid metabolism	0.91492	0.93286	0.07129
Sphingolipid metabolism	0.91492	0.93286	0.01288
Linoleic acid metabolism	0.93486	0.93486	0.65625

Table S-4. Clinicopathologic characteristics of 85 OS patients in the Xena Target-OS Hub.

Characteristics	No. of Patients (%) (N=85)	Mean (SD) (N=85)
**Gender**		
Male	47 (55.3)	-
Female	37 (43.5)	-
Missing	1 (1.2)	-
**Age**	-	14.5 (4.83)
**Tumor site**		
Leg	76 (89.4)	-
Arm	6 (7.1)	-
Pelvis	2 (2.4)	-
Missing	1 (1.2)	-
**Tumor region**		
Proximal	22 (25.9)	-
Distal	28 (32.9)	-
Others	3 (3.5)	-
Missing	32 (37.6)	-
**Metastasis**		
Yes	21 (24.7)	-
No	63 (74.1)	-
Missing	1 (1.2)	-
**Metastasis site**		
Lung	16 (18.8)	-
Bone	1 (1.2)	-
Both	5 (5.9)	-
Missing	63 (74.1)	-
**Race**		
Black	7 (8.2)	-
White	51 (60.0)	-
Asian	6 (7.1)	-
Missing	21 (24.7)	-
**Stage**		
I/II	7 (8.2)	-
III/IV	6 (7.1)	-
Missing	72 (84.7)	-

Table S-5. Clinicopathologic characteristics of 10 OS patients with primary foci.

No. of patient	Gender	Age	Tumor site	Stage
1	Male	21	Pelvis	III(T3bN0M0 G3)
2	Female	17	Leg	IIb(T2N0M0 G3)
3	Male	15	Arm	IIb(T2N0M0 G3)
4	Male	27	Leg	IIa(T1N0M0 G3)
5	Female	15	Leg	IIb(T2N0M0 G3)
6	Male	8	Leg	IIa(T1N0M0 G3)
7	Male	18	Arm	IIa(T1N0M0 G3)
8	Male	42	Pelvis	III(T3bN0M0 G3)
9	Female	18	Leg	IIa(T1N0M0 G3)
10	Male	35	Leg	IIb(T2N0M0 G3)

Table S-6. Clinicopathologic characteristics of 7 OS patients with metastases.

No. of patient	Gender	Age	Tumor site	Stage
1#	Male	63	Right lung	IV
2#	Male	18	Both lungs	IV
3#	Female	65	Right lung	IV
4#	Male	12	Left lung	IV
5#	Male	20	Left lung	IV
6#	Female	19	Right lung	IV
7#	Male	35	Left lung	IV

Table S-7. PSAT1 positive cell ratio and PSAT1 positive cell density in 7 pairs of samples.

	PSAT1 positive cell ratio（%）							P value
No. of patient	1#	2#	3#	4#	5#	6#	7#	
Tumor	71.68	60.11	23.01	25.45	29.67	26.40	32.59	0.043
Normal	29.86	24.69	15.86	17.43	28.60	5.09	37.19	
	PSAT1 positive cell density							P value
No. of patient	1#	2#	3#	4#	5#	6#	7#	
Tumor	0.1002	0.1581	0.0910	0.1116	0.0869	0.1234	0.0934	0.043
Normal	0.0993	0.0864	0.0970	0.0799	0.0801	0.0555	0.0741	
